# Glucose Starvation Alters Heat Shock Response, Leading to Death of Wild Type Cells and Survival of MAP Kinase Signaling Mutant

**DOI:** 10.1371/journal.pone.0165980

**Published:** 2016-11-21

**Authors:** Nora Plesofsky, LeeAnn Higgins, Todd Markowski, Robert Brambl

**Affiliations:** 1 Department of Plant Biology, University of Minnesota, Saint Paul, Minnesota, United States of America; 2 Department of Biochemistry, University of Minnesota, Saint Paul, Minnesota, United States of America; University of California Riverside, UNITED STATES

## Abstract

A moderate heat shock induces *Neurospora crassa* to synthesize large quantities of heat shock proteins that are protective against higher, otherwise lethal temperatures. However, wild type cells do not survive when carbohydrate deprivation is added to heat shock. In contrast, a mutant strain defective in a stress-activated protein kinase does survive the combined stresses. In order to understand the basis for this difference in survival, we have determined the relative levels of detected proteins in the mutant and wild type strain during dual stress, and we have identified gene transcripts in both strains whose quantities change in response to heat shock or dual stress. These data and supportive experimental evidence point to reasons for survival of the mutant strain. By using alternative respiratory mechanisms, these cells experience less of the oxidative stress that proves damaging to wild type cells. Of central importance, mutant cells recycle limited resources during dual stress by undergoing autophagy, a process that we find utilized by both wild type and mutant cells during heat shock. Evidence points to inappropriate activation of TORC1, the central metabolic regulator, in wild type cells during dual stress, based upon behavior of an additional signaling mutant and inhibitor studies.

## Introduction

The universal response of cells to high, non-lethal temperatures is characterized by the massive, but transient synthesis of heat shock proteins (Hsps) that protect cellular proteins from denaturation, help refold them, or alternatively guide their destruction [1–2]. This response is also characterized by increased reliance on glycolysis for energy generation [3] at the expense of mitochondrial oxidative respiration. We have asked whether cell recovery from heat shock could be compromised if glycolysis simultaneously was blocked by glucose starvation, induced by a glucose metabolism inhibitor, 2-deoxyglucose (2-DG) [4]. Indeed, we found that these conditions of dual stress (DS) proved lethal to wild type (wt) *Neurospora crassa*.

We reported earlier [4] that inhibition of fatty acid synthesis, particularly ceramide synthesis, restored survival to wt cells under DS; and we identified a novel long chain phytoceramide, made uniquely by vulnerable wt cells, that might signal cell death. In the present study we show that wt survival of DS is dramatically increased by addition of the anti-oxidant glutathione to the growth medium, implicating oxidative stress as a chief source of lethality. In support of this, our assays on intact cells demonstrate that wt has elevated reactive oxygen species (ROS) as a result of DS, but not in response to heat shock (HS). Similarly, whole cell assays show that nitric oxide (NO) peaks in wt during DS, but not during HS.

Furthermore, addition of rapamycin, in this study, protected wt cells from DS-induced death. Rapamycin inhibits the Target of Rapamycin Complex 1 (TORC1), which is the central driver of cell growth and anabolic metabolism under favorable conditions [5]. TORC1 inactivation by nutritional deficiency or heat shock, an effect mimicked by rapamycin addition, typically results in the autophagic degradation and recyling of cellular components [6], thereby enhancing survival under stress conditions.

In our earlier study of DS [4], we also assayed survival of the *os2* mutant strain, which lacks a functional stress MAP kinase [7]; we were prompted by reports that the p38 mammalian homolog participates in cell death signaling. The Os2 MAP kinase of *N*. *crassa*, like its homolog Hog1 in *Saccharomyces cerevisiae*, is activated by HS [4], as well as by hyperosmotic stress; and it contributes to the heat shock response, although it is not required for HS survival [7]. In contrast to the extremely low survival of wt cells subjected to DS, we found that cells lacking a functional Os2 or deleted in *os2* survive DS well [4], indicating that activation of Os2 is detrimental to cells that undergo DS. In the current report, we learned that the *os2* mutant strain (os2) does not show the DS-induced increase in ROS found for wt and only a small increase in NO.

To gain a wider understanding of changes in gene expression induced by HS and DS, we employed global assays of proteins and mRNAs present in extracts of wt and os2 cells, utilizing iTRAQ [8] and RNAseq protocols [9]. We have focused our analysis on metabolic pathways and on the regulatory mechanisms utilized by wt and os2, employing a variety of experimental approaches in addition to bioinformatics. The results suggest that wt and os2 cells undergo autophagy in response to HS, but that only os2 undergoes autophagy during DS. We propose that TORC1 may be inappropriately activated in wt undergoing DS. Possible signaling pathways are examined and discussed.

## Results

### 2-DG Uptake

We verified that wt and os2 cells under stress incorporated similar amounts of 2-DG by providing them with radiolabeled 2-DG and measuring the radioactivity associated with cell pellets and the residual radioactivity of the growth medium. This showed that the kinetics of uptake were slower in os2, so that within the first 15 min os2 cells had taken up 7% of the available labeled 2-DG and wt had taken up 37%. However, the difference in uptake diminished by 75 min, when 48% of the 2-DG had been taken up by os2 and 59% by wt. Furthermore, by utilizing liquid chromatography-mass spectrometry we performed an untargeted metabolomics analysis of wt and os2 cells at 30°C, HS, and DS [10]. We learned that only the DS treatment of the two strains produced extracts with very highly increased levels of 1-*O*-phosphonohexitol, with a ratio of 86 for wt DS/30°C (p value of 0.0128) and a ratio of 452 for os2 DS/30°C (p value of 0.0056). This metabolite likely represents a product of accumulated phosphorylated glucose, from glycogen degradation and other sources, whose further metabolism is blocked by the 2-DG incorporated into these cells.

### Cell Survival

**Fig 1:**
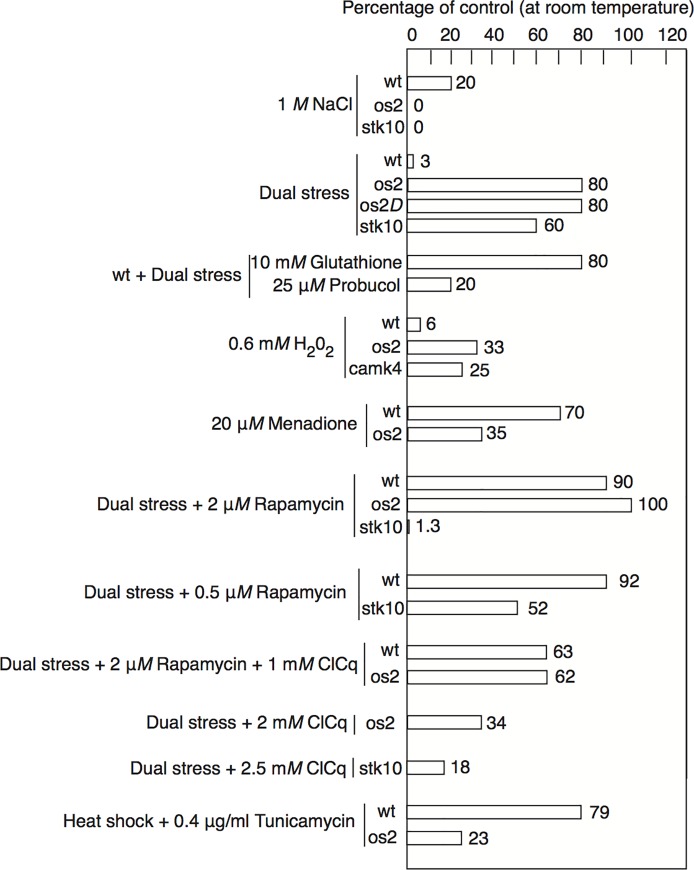
Percentage of surviving colonies, representing viable conidiospores, in petri plate assays. The controls in each case consist of the particular strain incubated at room temperature. At least three plates were counted for each treatment, and experiments were performed at least two times with similar results. Typical examples of reproducible experiments are shown.

To detect possible signaling proteins involved in susceptibility to DS, we measured the DS survival of selected deletion mutant strains, which were developed through the *Neurospora* Genome Project [11] and provided by the Fungal Genetics Stock Center [12]. We learned that the *os2* deletion mutant survived 80% under DS, but the os2 strain that we employed in further experiments for this study is a long-studied point mutant strain, UCLA 80, that produces a truncated non-functional protein [13]. This mutant strain also shows high survival in response to DS, varying between 75% and 90% of its control (Fig 1), whereas wt is largely killed by DS, surviving 2% to 5% of its room temperature (RT) control in petri plate assays.

Most other assayed strains, mutant in signaling pathways, such as mek1, mak1, and camk4, showed a low survival similar to that of wt, but a few showed more resistance to DS. Notably, when we assayed stk10, lacking the *Neurospora* gene homologous to *S*. *cerevisiae sch9*, we found that it survived DS 50% to 70% of its control (Fig 1). Deletion of genes for *sch9* extends replicative lifespan of yeast, mimicking the effects of calorie restriction [6].

We added compensatory or inhibitory reagents to the petri plate medium to determine whether they protected cells undergoing DS or replicated the damage of DS. The reduced tripeptide glutathione is a powerful anti-oxidant as well as a detoxifying agent [14]. We found that 10 mM glutathione consistently increased DS survival of wt to 75–80% of control (Fig 1), which is comparable to os2 survival without glutathione. 25 μM probucol, an anti-oxidant that protects lipids from peroxidation [15], reproducibly increased wt survival from 1% to 20% (Fig 1), supporting the more dramatic results with reduced glutathione.

Because these results pointed to oxidative stress as a major contributor to wt death under DS, we added pro-oxidants to RT petri plate assays of wt and os2. The addition of hydrogen peroxide, at various concentrations, showed that os2 had a 4- to 6-fold enhanced resistance to this oxidant, compared with wt (Fig 1). For example, 0.6 mM hydrogen peroxide resulted in wt survival of 6% and os2 survival of 33%. This resistance may not be relevant to os2 DS survival, however, since DS proved lethal to a camk4 deletion mutant that also showed greater resistance to hydrogen peroxide than wt (Fig 1). Furthermore, addition of the pro-oxidant menadione to cell assays at RT had a contrasting outcome to hydrogen peroxide. 20 μM menadione resulted in wt survival of 70% and os2 survival of 35%, a 2-fold difference (Fig 1); at different menadione concentrations the ratio was higher or lower, but always to the advantage of wt. This indicates that os2 does not have a greater intrinsic resistance to all forms of oxidative stress. In contrast to our findings, earlier studies [16] reported that the *os2* mutant strain was more inhibited by hydrogen peroxide than wt, according to the rate of colony extension. However, the developmental stage and type of measurements were different from those reported here.

Cellular processes affected by addition of 2-DG would be expected to include cell wall deposition and protein glycosylation, in addition to energy generation. Alterations in cell wall glycans likely activate protein kinase c (Pkc) and the protective cell integrity MAP kinase cascade. In Western blots, discussed below, we found that DS increased the amount of phosphorylated Mak1 of this pathway above control levels in both wt and os2, but Mak1 appeared hyperphosphorylated in os2. To stimulate the cell wall integrity (CWI) pathway further in wt cells during DS, we added to them 40 μM dioctanoyl-glycerol, an analog of diacylglycerol that stimulates Pkc [17]. Although this concentration should be in the biologically effective range, it had no effect on wt survival (data not shown). Another likely effect of 2-DG is the disturbance of sugar modifications on membrane and secreted proteins. As an analog, we added the protein glycosylation inhibitor tunicamycin at 0.4 μg ml^-1^ to wt and os2 cells during HS. We found that wt survival was 79% and that of os2 was 23% (Fig 1), almost a 4-fold difference; at 0.8 μg ml^-1^ there was lower survival and a 10-fold difference between strains. Therefore, the DS survival advantage of os2 over wt does not derive from tolerance for disturbed glycosylation of proteins.

Rapamycin is an inhibitor of TORC1 [6], the kinase complex that is central to cell growth and proliferation under favorable conditions. Inactivity of TORC1 under unfavorable conditions slows growth and promotes mechanisms such as autophagy that allow cell survival. Like deletion of *sch9* or *tor1*, rapamycin addition enhances life span extension in yeast [6]. We found that 2 μM rapamycin added to plate assays fully restored DS survival to wt and os2 cells (Fig 1). All strains that we treated with 2 μM rapamycin during DS showed partially increased survival, including *atg1* and *atg7* deleted strains, suggesting that rapamycin may enhance survival in ways additional to autophagy. The exception to this enhanced survival was stk10/sch9, whose survival under DS declined from 50% without rapamycin to 1.3% with rapamycin. Only a lower amount of rapamycin, 0.5 μM, failed to depress stk10 survival, while still completely protecting wt during DS. These results show that TORC1 inactivation counteracts the damaging effects of DS, while they also suggest that TORC1 activity may be at least partially disabled already in the stk10 strain, requiring calibration of rapamycin to avoid excessive, damaging inhibition of TORC1.

The autophagy that results from TORC1 inactivity requires acidified vacuoles/lysosomes, and autophagy can be blocked by the anti-malarial drug chloroquine, which accumulates in vacuoles and alkalizes them [18]. When we added 1 mM chloroquine to rapamycin-treated cells undergoing DS, survival of both wt and os2 declined from 100% to 63% (Fig 1), suggesting that at least one beneficial effect of rapamycin addition and TORC1 inhibition is the enhancement of autophagy. Furthermore, in the absence of rapamycin, we found that 2 mM chloroquine brought os2 DS survival from 94% to 34%, and 2.5 mM reduced stk10 survival from 46% to 18% (Fig 1). This indicates that autophagy is likely an important source for cellular resistance to DS in these two mutant strains.

### Measurement of Reactive Oxygen and Nitrogen Species

**Fig 2:**
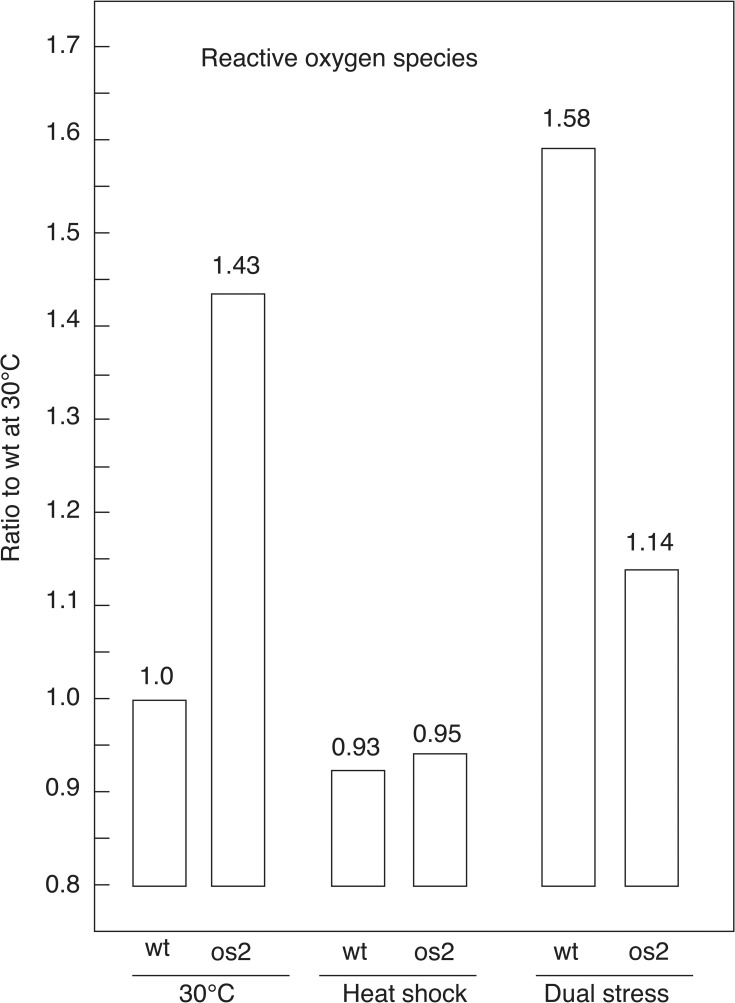
Ratio of reactive oxygen species (ROS) in wt and os2, according to fluorescence measurements. Germinating conidiospores were incubated 1 hr at control (30°C), HS (45°C), or DS (45°C and 2-DG) conditions. The measurements are expressed as a ratio to those in the wt control. Experiments were performed at least two times with similar results.

The positive effect of glutathione on wt survival during DS suggests the presence of damaging reactive oxygen species and/or reactive nitrogen species in wt cells. To derive relative measurements for ROS and nitric oxide (NO) in intact cells, we employed cell-permeant, fluorescent reagents. For ROS detection, 2’,7’-dichlorodihydrofluorescein diacetate was added to wt and os2 cells during a 60 min exposure to HS, DS or continued incubation at 30°C. The cells were washed, diluted to four different concentrations in buffer, and aliquoted into microplate wells. Readings were recorded from a microplate reader, normalized, and averaged for each treatment. This showed that after one hr exposure to DS, the ROS signal for wt was 58% higher than its 30°C signal (Fig 2). In contrast, the ROS signal for os2 during DS was 20% lower than its 30°C signal, which is apparently elevated. Nevertheless, in the first hr of DS, wt has 39% higher levels of ROS than os2 has. When their ROS signals during HS are compared, wt and os2 are equivalent and similar to wt 30°C levels. These results show that ROS are not elevated during HS in either strain and that there are similar levels in wt and os2 during HS. In contrast, ROS are elevated in wt during DS both in comparison to its 30°C level and in comparison to the os2 DS level. For os2, the anti-oxidants induced by either stress treatment likely reduce ROS below their elevated 30°C levels, but the HS level is lower than the DS level.

**Fig 3:**
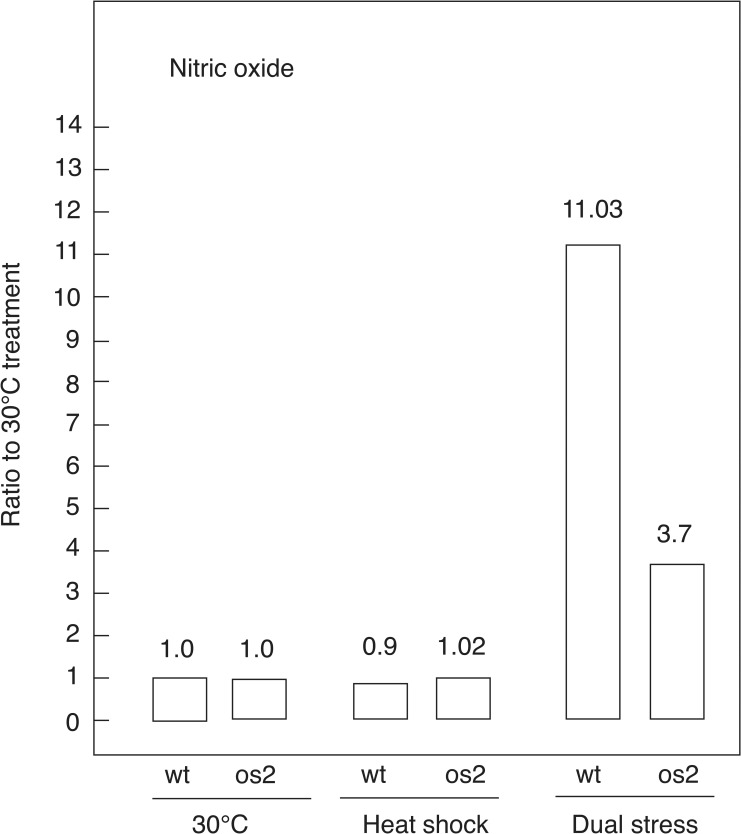
Ratio of nitric oxide (NO) in wt and os2, according to fluorescence measurements. Germinating conidiospores were incubated 1 hr at control (30°C), HS (45°C), or DS (45°C and 2-DG) conditions. The measurements are expressed as a ratio to the control of each strain. Experiments were performed at least two times with similar results.

To detect NO in intact cells, we added 4-amino-5-methylamino-2’,7’-difluorofluorescein diacetate during the last 30 min of a 60 min exposure to HS, DS or continued incubation at 30°C; and we utilized the same general procedures used to measure ROS. Unlike with ROS, the 30°C levels of NO were similar for the two strains, with that of os2 being only 8% higher than in wt. Furthermore, both strains showed an increase in NO as a result of DS treatment. However, the increase over 30°C levels was much greater for wt than for os2; there was a 9- to 16-fold increase for wt, with an average of 11-fold, compared with a 3- to 4-fold increase for os2 (Fig 3). In contrast, HS led to a 10% decline from the 30°C NO signal in wt and no change for os2. These results indicate that combining 2-DG with HS produces a large increase in NO over 30°C or HS alone and that this increase is especially high for wt.

### Bioinformatics Analysis

**Fig 4:**
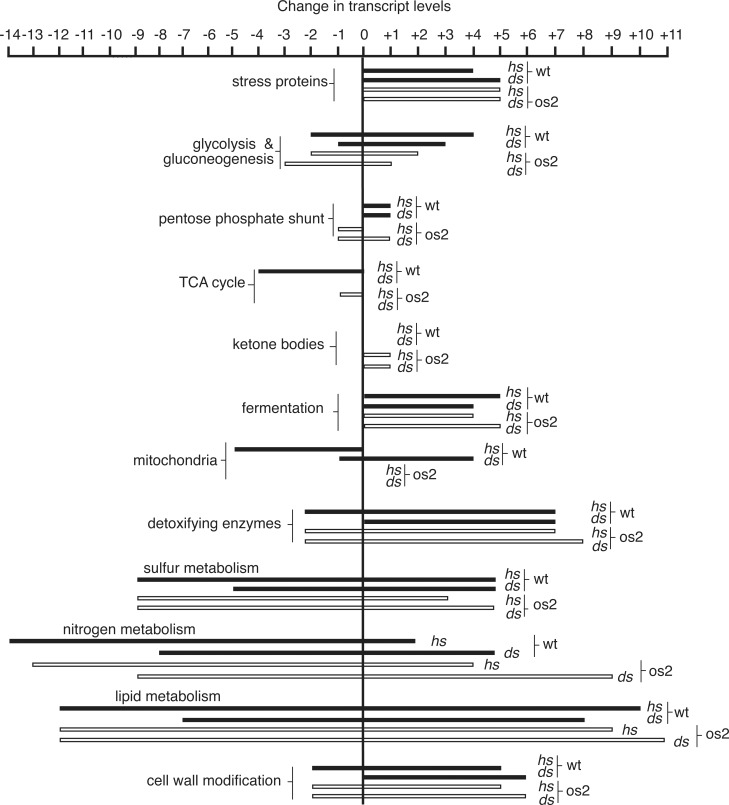
Categories of transcripts whose levels increase or decrease during stress: Metabolism. Numbers of up- or down-regulated transcripts discussed in this report, are indicated for wt (filled bars) and os2 (outlined bars) during heat shock (hs) or dual stress (ds). For details of transcript levels for individual proteins see Supporting Information.

**Fig 5:**
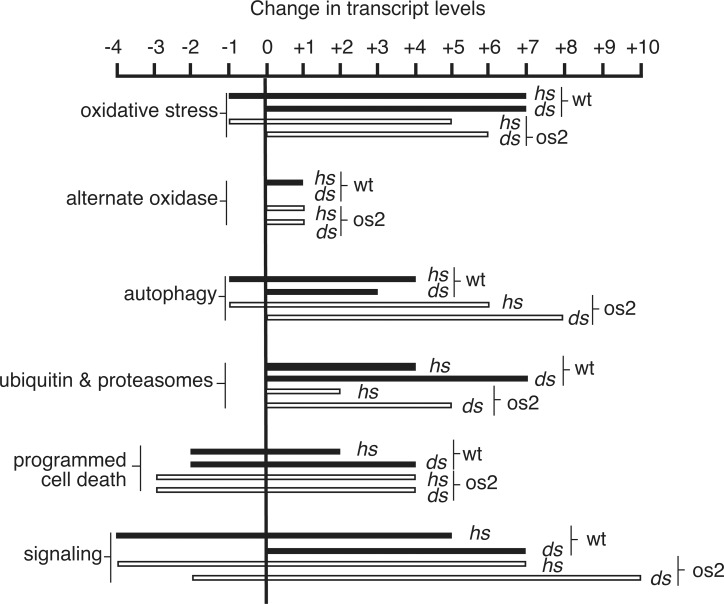
Categories of transcripts whose levels increase or decrease during stress: Signaling. Numbers of up- or down-regulated transcripts discussed in this report, are indicated for wt (filled bars) and os2 (outlined bars) during heat shock (hs) or dual stress (ds). For details of transcript levels for individual proteins see Supporting Information.

iTRAQ protein comparisons were made between wt and os2 after three hr of exposure to DS conditions (Table 1, S3 Table). Proteomics provides a picture of the detected proteins present in cells, as well as the ratio between mutant and wt strains for particular proteins. We also employed RNAseq to compare transcripts in wt and os2 cultures exposed to three conditions: 30°C control, two hr of HS, or two hr of DS (Figs 4 and 5, S1 and S2 Tables). RNAseq yields approximate quantities of transcripts, as well as transcript ratios between different treatments. Given the variables of transcript stability and translational efficiency, however, it is not as directly related to cellular function as proteomics. The proteins and transcripts detected in cells subjected to DS may derive either from the single stresses of heat shock or glucose-deprivation or from the combined stresses.

**Table 1:** iTRAQ Protein Ratios os2/wt During Dual Stress.

NCU#	protein	ratio	p value	NCU#	protein	ratio	p value
	**STRESS**				**OXIDATIVE**		
NCU09364	Hsp30	1.57	0.006	NCU08791	Catalase 1	0.82	0.030
NCU04142	Hsp90	1.52	1.10E-22	NCU00355	Catalase 3	0.75	0.079
NCU09602	Hsp70	1.32	0.018	NCU05169	Catalase 4	0.56	0.002
NCU05269	Hsp88	1.20	1.58E-09	NCU03339	Glutath Red	0.82	0.000
NCU07414	Ydj1	1.14	0.025	NCU06556	Thioredox 2	0.76	0.008
NCU03732	Sis1	1.13	0.032	NCU02595	Thioredoxin	0.82	0.023
NCU01792	p23	1.37	0.061	NCU04098	Glutaredox 5	0.84	0.165
NCU00714	Sti1	1.31	3.75E-09	NCU06031	Prx1 MitoPer	0.89	0.014
NCU04172	Fes1	1.11	0.097	NCU05770	Catalase 2	1.52	8.61E-12
NCU03982	Grp78	1.17	3.99E-06	NCU09560	Fe/Mn Sod	1.37	0.070
NCU02630	Hsp78	1.17	0.003	NCU07851	Sod1 cu chap	1.21	0.190
NCU01589	Hsp60	1.10	0.001	NCU09040	Mig4	1.36	7.84E-06
NCU04334	Hsp10	1.11	0.094	NCU01759	Mig5	1.25	0.016
NCU03853	Cyp40	1.42	7.26E-07	NCU09210	DyP-Type Per	1.33	0.110
NCU01200	CypB	0.84	0.026	NCU03151	Peroxis Per	1.11	0.206
NCU02455	FKBP22	0.78	0.015		**AUTOPHAG**		
NCU01516	GrpE	0.83	0.019	NCU01955	Atg3	1.14	0.200
	**GLYCOLYS**			NCU00673	Spr4 Ser Prot	1.53	0.042
NCU06075	Pyruvate Kin	1.12	0.005	NCU04192	Lap4	1.15	0.026
NCU10042	Enolase	1.09	0.007	NCU11129	Carboxypep s	1.39	0.083
NCU02542	Hexokinase	0.91	0.032	NCU02273	Pep4	1.15	0.107
NCU07550	Triose-P Iso	0.88	0.018	NCU07020	Vps17	1.16	0.152
NCU07281	Gluco 6-P Iso	0.88	0.001	NCU03463	Vac ATPase 1	1.10	0.061
	**GLUCONEO**			NCU16822	Peptidase Inh	0.81	0.015
NCU09873	PEP Carboxy	1.24	9.50E-05	NCU08677	Peptidase Inh	0.68	0.011
	**PENT PP**			NCU06666	Inositol3P Syn	1.44	5.83E-10
NCU02136	Transaldol	0.88	0.001		**UBIQUITIN**		
NCU01328	Transketol	0.85	4.91E-06	NCU06712	Pre5	1.17	0.181
NCU03100	6PGluconDH	0.82	7.08E-06	NCU03028	Dph1	1.29	0.030
NCU00519	RibulP3Epi	0.77	0.045	NCU01919	Ubiq C-Hydro	1.12	0.036
NCU03651	NADPMalicE	0.65	0.091		**CELL DEATH**		
	**TCA CYCLE**			NCU01419	QuinOxiR Pig3	0.77	0.049
NCU08471	SuccCoAligβ	1.09	0.006	NCU05850	AIF	0.59	1.16E-09
NCU03004	PyruvDH E1	1.10	0.109	NCU06061	AMID	1.43	0.068
NCU00596	Lipoyltransf	1.25	0.120	NCU04452	Mig3 Oye	1.09	0.047
NCU02481	2MethylisLya	1.12	0.137		**SIGNALING**		
	**KETONES**			NCU07024	Os2	0.19	0.009
NCU05419	OHmeglutCo	1.16	0.065	NCU02815	Os1	0.90	0.154
NCU06881	SucCoAketo	1.35	0.080	NCU04600	Phosphat2C	0.88	0.063
NCU09553	OHbutCoDH	1.16	0.088	NCU06419	Mek1	0.80	0.033
	**MITO ETC**			NCU07495	Lsp1	0.47	1.98E-05
NCU00969	NADHDH17.8	1.62	0.001	NCU02540	Pil1	0.68	0.002
NCU02280	NADHDH21.3	1.35	0.036	NCU09212	Camk4	0.43	0.002
NCU16028	Cox2	1.24	0.026	NCU07966	CaATPase3	1.60	0.014
NCU05457	Cox4	1.23	0.026	NCU05046	CaATPase3like	1.21	0.005
NCU06695	Cox6	1.22	0.108	NCU09043	Caleosin Dom	1.28	0.023
NCU06741	Cox6b	1.12	0.064	NCU00326	Regucalcin	0.64	0.137
NCU09816	Cyt1	1.23	0.002	NCU00472	Cdc37	1.33	3.09E-06
NCU06748	Coa1	2.03	0.002	NCU02800	Ars2	1.16	0.033
NCU02623	Rcf2	1.62	0.081	NCU03087	ChpA	1.18	0.005
NCU03147	Cbp4	1.33	0.082	NCU00685	Casein Kin 1	1.20	0.088
NCU02267	Fmp25	1.55	0.050	NCU05810	Cpc2	0.81	0.001
	**ALTOX-RED**				**SULFUR**		
NCU08980	Nde2	1.29	0.000	NCU10016	ThermoDesulf	7.15	8.37E-06
NCU03112	NADHCytb52	1.20	5.87E-05	NCU05340	AlkSulfMono	4.68	0.053
NCU04443	Ndh2	1.25	0.086	NCU07819	TaurineDiox	4.76	5.06E-07
NCU00216	NADHCytb51	0.89	0.126	NCU07610	TaurineDiox	2.17	0.019
	**MITO**			NCU01057	TaurineDiox	2.10	0.020
NCU00894	Mitofilin	1.17	0.001	NCU01652	AcetHomoser	0.89	0.136
NCU08946	Phb1Prohib	1.28	1.60E-05	NCU05340	MetSynthase	0.90	0.015
NCU03310	Prohibitin 2	1.12	0.037	NCU09230	MetSynthase	0.89	1.80E-05
NCU05313	Fission 1	1.22	0.184	NCU07112	MetSynthase	0.45	6.47E-05
NCU02064	ApoO-Like	1.29	0.001	NCU06228	EnolPhosphE1	0.79	0.056
NCU04304	Porin	1.11	0.008	NCU08434	S-AdMetSyn	0.90	0.014
NCU00227	CationTransp	1.90	0.005	NCU07690	MeTetrahyFo	0.83	0.000
NCU07263	CarnitineCarr	1.85	0.015	NCU02274	SerOHmetran	0.88	0.000
NCU10732	DicarboxTran	3.13	0.034	NCU09230	Cys γ-Lyase	1.20	0.020
NCU08561	SuccFumTran	1.35	0.025	NCU07112	SulfQuinOx	1.10	0.038
NCU01689	Yhm2	1.12	0.041	NCU04636	CysDesulf	0.89	0.056
NCU04945	Mia40	1.17	0.135	NCU02007	Isd11	0.84	0.169
NCU03359	Iap1AAA Prot	2.65	5.47E-06		**NITROGEN**		
NCU00030	Nuclease	1.24	0.073	NCU05298	NitrateRed	0.77	3.77E-05
NCU03297	Cyt c Perox	1.38	1.51E-06	NCU04720	NitriteRed	0.82	0.000
NCU01501	Pth2PepRNA	1.26	0.024	NCU02361	Formamidase	0.60	5.10E-05
NCU09999	RibProL9Like	0.86	0.027	NCU03949	NitroproDiox	1.36	0.049
NCU12023	37SRib MRP4	0.85	0.048	NCU04856	GlnSynthet	0.84	0.016
NCU04806	RibProtein S7	0.82	0.026	NCU06724	GlnSynthet	0.79	0.023
NCU00395	Mss51-Like	0.87	0.056	NCU06803	GluDecarbox	0.74	0.001
NCU02955	G1 Transl EF	0.89	0.023	NCU06112	GluDecarbox	0.76	0.088
NCU09331	Hmf1/Mmf1	0.77	0.019	NCU04303	Uricase	0.78	0.042
	**LIPIDS**			NCU00414	AdenosineKin	0.87	0.011
NCU08828	PeroxFAOx2	0.89	0.001	NCU05542	XanthRibosyltr	0.89	0.181
NCU00742	NADgl3P DH	0.90	0.045	NCU06300	GuanylateKin	0.88	0.059
NCU04923	Glycerol DH1	0.71	5.14E-06	NCU03813	ArgSuccLyase	1.14	0.004
NCU04510	Glycerol DH3	0.84	0.004	NCU07853	AcOrnithDeAc	1.27	0.095
NCU03779	DiOHaceKin	0.78	0.044	NCU06727	Spe3	1.11	0.082
NCU03779	Glycero3PDH	1.27	1.91E-05				

### Stress Proteins: Bioinformatics

At the transcriptional level the heat shock response of wt is stronger than that of the *os2* signaling mutant, with stress-specific transcripts being induced to higher levels in wt and normal transcripts being dramatically reduced (S1 and S2 Tables). The greatist increase is seen for Hsp30 transcripts, which are elevated 187-fold in wt and 64-fold in os2 over control levels. When carbohydrate deficiency is added to heat stress, both wt and os2 increase their stress-specific transcripts, with wt achieving very high levels. This DS-induced increase in Hsp30 transcripts is 1,376-fold in wt and 557-fold in os2. It is notable, however, that normal transcripts appear relatively stable in wt under DS, with only moderate reductions for some and small increases for others. In contrast, os2 displays larger transcript decreases during DS than it does during HS (S2 Table). This suggests that os2 may perceive DS as an enhanced stress, and wt may perceive HS and nutritional deprivation as conflicting stresses. There is also the unexplored possibility that 2-DG or the accumulated hexose metabolite is perceived as a high glucose signal by wt, but not by os2.

Proteomics analysis, which provides a ratio between DS-treated os2 and wt (os2/wt) for detected proteins, shows that at three hr os2 is higher in all the major Hsps (Table 1). This is in strong contrast to Hsp transcript levels at two hr of DS (S1 Table). These proteins include Hsp30 (1.57), Hsp90 (1.52), Hsp70 (1.32), Hsp88 (1.20), and the J-proteins Ydj1 (1.14) and Sis1 (1.13). os2 is also higher in the Hsp70 nucleotide exchange factor, Fes1 (1.11*), and in the Hsp90 co-chaperones p23 (1.37*) and Sti1 (1.32). The organellar Hsps (Table 1) and cytosolic cyclophilin-40 (1.42) are also increased in os2, but the endoplasmic reticulum (ER)-localized immunophilins are decreased in os2 relative to wt. Nevertheless, the lower abundance of major Hsps in wt than in os2 during DS suggests that wt transcripts may become unstable, their translation may be impaired, or extensive proteolysis may be occurring in wt. We believe that the wt translational apparatus may become unregulated by DS, since wt transcripts for ribosomal and ribosomal processing proteins are relatively stable during DS, in contrast to their strong reduction during HS (S2 Table). These transcripts in os2 are depressed similarly during HS and DS.

### Carbohydrate Metabolism: Bioinformatics

#### Glycolysis

In response to high temperature, carbohydrate metabolism changes from a focus on oxidative respiration to an increase in glycolysis and fermentation [2–3]. However, addition of an inhibitor of glycolysis, such as 2-DG, would be expected to shift the balance from intense glycolysis to increased oxidative respiration. Stage-specific differences between the two strains in their use of glycolysis during DS are suggested by proteomics (Table 1). os2 is moderately high in pyruvate kinase (1.12) and enolase (1.08) of the later ATP-generating phase of glycolysis, but it is moderately low in enzymes of the initial ATP-utilizing phase, a hexokinase variant (0.91), triosephosphate isomerase (0.88) and glucose 6-phosphate isomerase (0.88).

#### Fermentation

According to increased transcripts for fermentative enzymes (Fig 4, S1 Table), however, wt and os2 both utilize mixed acid and pyruvate fermentation to metabolize carbohydrates and generate energy during HS and DS. Proteomics analysis (S3 Table) indicates that os2 is higher than wt during DS in four enzymes of ethanol fermentation [19], but it is lower than wt in the major fermenting alcohol dehydrogenase I (0.87).

#### Gluconeogenesis

Stress leads to increased transcripts for PEP carboxykinase (Fig 4, S1 Table), which are elevated 3.3-fold in each strain during DS. Proteomics (Table 1) show that os2 has higher amounts than wt of this enzyme (1.24), which catalyzes the rate limiting step in gluconeogenesis, suggesting that os2 is more active in gluconeogenesis than wt during DS.

#### Pentose Phosphate Pathway

The pentose phosphate pathway [20], running parallel to glycolysis, generates 5-carbon sugars and NADPH for molecular biosyntheses and reducing agents. By proteomics (Table 1), os2 is low in several enzymes of the pentose phosphate pathway, including transaldolase (0.88), transketolase (0.85), 6-phosphogluconate dehydrogenase (0.82), and ribulose-phosphate 3-epimerase (0.77). Transcripts for transaldolase decrease in os2 during stress (S2 Table).

#### TCA Cycle

Proteomics (Table 1) suggest that os2 is more active than wt in the TCA cycle during DS, since os2 is moderately higher in two TCA enzymes, a succinyl-CoA ligase subunit (1.09) and a pyruvate dehydrogenase E1 component (1.10*). Furthermore, the lipoyltransferase that is essential for modifying and activating many oxidative ezymes, notably TCA cycle pyruvate dehydrogenase, is upregulated in os2 over wt (1.25^§^).

#### Ketone Bodies

High levels of acetyl-CoA, resulting from catabolism, can accumulate in mitochondria when its entry into the TCA cycle is blocked by a shortage of oxaloacetate. This leads to the formation of ketone bodies such as acetoacetate, which can later be catabolized back to acetyl-CoA [21]. Proteomics analysis (Table 1) shows that os2 is higher than wt in multiple enzymes involved in ketone body formation and dissolution, including hydroxymethylglutaryl-CoA lyase (1.16*), a succinyl-CoA:3-ketoacid CoA-transferase subunit (1.35*), and 3-hydroxybutyryl-CoA dehydrogenase (1.16*). Transcripts for hydroxymethylglutaryl-CoA lyase accumulate to 3.4-fold higher levels in os2 during both HS and DS, compared with 30°C (S1 Table).

#### Glycogen

HS and DS lead both strains to increase transcripts for glycogen phosphorylase and glucoamylase, enzymes that degrade glycogen, the main storage form of glucose (S1 Table). Proteomics analysis (S3 Table) shows that os2 has higher amounts of glycogen phosphorylase (1.15) and glycogen debranching enzyme (1.14), as well as the starch degrading enzyme α-amylase (1.37).

### Mitochondria: Bioinformatics

#### Energy-Yielding Electron Transport

Although mitochondrial oxidative respiration is the most efficient way to generate energy from limited glucose resources, it can also be a source of damaging ROS when electron flow is inhibited [22]. During HS both strains, especially wt, have depressed levels of transcripts for electron transport chain components (Fig 4, S2 Table). In contrast, under DS wt transcripts for subunits of cytochrome *c* oxidase, the cytochrome *bc*_1_ complex, and ATP synthase are stable or even increased over control levels. Cytochrome *c* mRNA is especially dramatic, declining in wt 16.7-fold in response to HS, but increasing approximately 2-fold from control during DS. Levels of these transcripts are approximately 3.5-fold lower in os2 compared with wt. Despite higher levels of transcripts in wt for respiratory enzymes, proteomics (Table 1) show that os2 is higher than wt in subunits of NADH dehydrogenase (1.62 and 1.35), cytochrome *c* oxidase (1.24, 1.23, 1.22*, and 1.12*), and cytochrome *c*_1_ of the *bc*_1_ complex (1.23). os2 is also higher in assembly proteins for respiratory complexes (Table 1), including Coa1 (2.03) and Rcf2 (1.62*) for cytochrome *c* oxidase and Cbp4 (1.33*) and Fmp25 (1.55) for Cyt *bc*_1_.

#### Alternative Oxidoreductases

Mitochondrial electron transport may be maintained by cells during stressful or inhibitory conditions, even without ATP synthesis, to minimize formation of reactive oxygen species (ROS) (22). One mechanism is to utilize alternative oxidoreductases that transfer electrons without generating ATP. Alternative oxidases (Aods) transfer electrons from ubiquinone directly to oxygen without yielding energy [23]. We found that Aod3 transcripts are increased 18.7-fold by DS in os2 only (S1 Table). In proteomics analysis (Table 1), os2 has higher amounts than wt of alternative oxidoreductases that circumvent complex I of the respiratory chain, which is a major source of mitochondrially generated ROS [24]. These oxidoreductases include the Nde2 external NAD(P)H dehydrogenase (1.29), NADH cytochrome *b*_*5*_ reductase 2 (1.20), and Ndh2 NADPH_2_-quinone oxidoreductase (1.25*). Transcripts for the inner membrane NAD(P)H dehydrogenase Nde1 [25] increase 3.7-fold in os2 during DS compared with HS.

#### Other Mitochondrial Functions

Proteins that help organize the inner mitochondrial membrane are elevated in os2 during DS, compared with wt (Table 1). These include mitofilin (1.17), prohibitin-like proteins (1.28 and 1.12), and the mitochondrial fission 1 protein (1.22^§^). os2 also has higher amounts of an apolipoprotein O-like protein (1.29) that, in mammals, binds to mitofilin and affects cristae morphology [26]. Furthermore, os2 has high levels of several mitochondrial transport proteins (Table 1), the AAA protease IAP1 (2.65), a mitochondrial nuclease (1.24*), and the anti-oxidant cytochrome *c* peroxidase (1.38).

Although the mitochondrial Pth2 peptidyl-tRNA hydrolase (1.26), which facilitates tRNA recycling for translation, is more abundant in os2, it is lower than wt in many mitochondrial ribosomal proteins (0.86–0.82) and in Mss51 (0.87) the Cox1 mRNA translational activator [27]. It is also lower in the G1 translation elongation factor (0.89) and in Mmf1 (0.77), which interacts in *S*. *cerevisiae* genetic screens with mitochondrial ribosomal proteins [28]. Induction of *mmf1* by stress in yeast is dependent on Hog1 [29], the homolog of Os2. Despite these indications that mitochondrial translation may be reduced in os2, the mitochondrially encoded Cox2 subunit of cytochome *c* oxidase (1.24) is higher in os2 than in wt (Table 1).

### Oxidative Stress: Bioinformatics

Proteomics (Table 1) show that the major anti-oxidant proteins are higher in wt than in os2 during DS, with a few exceptions. wt is higher in catalase-1 (0.82), -3 (0.75*), and -4 (0.56); and it has higher amounts of many thiol-related proteins, such as glutathione reductase (0.82), thioredoxin 2 (0.76), a thioredoxin domain-containing protein (0.82) and mitochondrial glutaredoxin 5 (0.84^§^). On the other hand, os2 has higher amounts of catalase 2 (1.52), a mitochondrial (Fe/Mn) SOD (1.37*), a copper chaperone for cytoplasmic (Cu/Zn) SOD (1.21^§^), and the Mig4 (1.36) and Mig5 (1.25) reductases.

Many transcripts for anti-oxidant proteins increase during both HS and DS (Fig 5, S1 Table). These transcript increases are generally greater for wt than for os2. For example, catalase 2 transcripts strongly increase 100- to 125-fold in wt and 43- to 60-fold in os2. Catalase 1 mRNA increases 40-fold in wt during HS and 12-fold during DS, but only 3.7-fold in os2 during DS; it is a gene whose full induction by stress is reported to depend on Os2 [7].

### Autophagy and Vacuoles: Bioinformatics

Macro-autophagy (also referred to as autophagy) involves the vacuolar degradation and recycling of cellular constituents for cell maintenance and growth [30]. Since autophagy frequently functions as a protective response to starvation and other stresses, it might enhance resistance to DS, a proposal supported by experimental results presented above. Proteomics show that several vacuolar proteases are upregulated in os2 compared with wt (Table 1). The highest in os2 is a subtilisin-like serine protease (1.53), whose vacuolar Prb1 homolog is induced in yeast by starvation and sporulation [31]. Three other vacuolar proteases that are elevated in os2 are Lap4 (1.15), carboxypeptidase s (1.39*), and Pep4 (1.15*), which processes and activates other vacuolar proteins. os2 also has higher content of the vacuolar sorting protein Vps17 (1.16^§^) and the vacuolar pH-sensitive ATPase (1.10*). Two proteins with peptidase inhibitor domains (0.81 and 0.68) are reduced in os2. Transcripts for several vacuolar sorting and transport proteins are also uniquely elevated in os2 during DS, including mRNAs for vacuolar sorting receptors, vacuolar amino acid transporter 1, and oligopeptide transporter 2 (S1 Table).

Many autophagy-related proteins participate in the formation of autophagosomes that deliver proteins and other cellular constituents to the vacuoles/lysosomes for degradation. Compared to wt, os2 is enriched in the E2-like autophagy protein Atg3 (1.14^§^), involved in protein lipidation and phagophore expansion [32] (Table 1). Transcripts for certain autophagy proteins are upregulated during both HS and DS, but a greater number of them have 3- to 12-fold increased transcripts in os2 during DS (Fig 5, S1 Table). These include transcripts for the Atg1 kinase [30, 32], an essential, early autophagy protein, Atg17, an early scaffold protein of pre-autophagosomes, and Atg18 [32], which binds to phosphatidylinositol 3-phosphate, a membrane lipid that is essential for autophagosome biogenesis. These transcript comparisons suggest that autophagy may be a component of the protective heat shock response of wt that is not utilized during DS, whereas autophagy is utilized by os2 during its DS response.

### Ubiquitin and Proteasomes: Bioinformatics

Ubiquitin-modification can mark proteins for proteolysis by the 26S proteasome, which is responsible for non-vacuolar degradation of denatured, damaged, or short-lived proteins [33]. Most ubiquitin-related transcripts are particularly elevated during DS, compared with HS, in both wt and os2 (S1 Table). These DS-induced transcripts encode polyubiquitin, a ubiquitin ligase, a 26 S proteasomal subunit, a RING-10 ubiquitin hydrolase, and two ubiquitin fusion degradation proteins. According to proteomics (Table 1), os2 is higher than wt in a few ubiquitin/proteasome-associated proteins that include the Pre5α-6 proteasomal subunit (1.17^§^); Dph-1 (1.29), an ubiquitin-binding protein that protects against de-ubiquitination; and an ubiquitin C-terminal hydrolase (1.12) that de-ubiquitinates proteins. The ubiquitin-proteasomal system is clearly important to both wt and os2 for degrading denatured and damaged proteins during DS.

### Programmed Cell Death: Bioinformatics

Apoptosis or programmed cell death (PCD) typically involves a cascade of proteolytic caspases that effect changes in membranes, chromatin, and overall cell morphology, ultimately leading to self-destruction [34]. Although the machinery of PCD is not well defined in mycelial fungi, it has been described for sphingolipid-induced death [4, 24], farnesol-induced apoptosis [35], and heterokaryon incompatibility [36]. Furthermore, *Neurospora* has proteins that resemble known apoptosis-related proteins in other biological systems.

The DS-induced death of wt depends upon ROS, which are an important hallmark of apoptosis [24]. Certain reductases and dehydrogenases in *Neurospora* have been identified as cell death-associated, being either pro- or anti-apoptotic. According to this identification, wt has higher amounts of pro-apoptotic proteins and os2 has higher amounts of anti-apoptotic proteins during DS (Table 1). The pro-apoptotic proteins include a NADPH-quinone oxidoreductase (0.77), similar to PIG3 [37], and rubredoxin-NAD+ reductase/AIF (0.59), whose gene deletion was reported to protect *Neurospora* cells from phytosphingosine-induced death [24]. On the other hand, os2 has higher amounts of a protective cytoplasmic NADH-dehydrogenase [24], identified as AMID (1.43*), and higher amounts of Oye2/Mig3 (1.09), an NADPH-dependent dehydrogenase, whose overexpression increased yeast resistance to apoptotic agents [38]. This imbalance of putative pro- and anti-apoptotic proteins favors the susceptibility of wt to PCD and the resistance of os2.

Transcript analysis gives some support to PCD being an important part of the stress response (Fig 5, S1 and S2 Tables). Transcripts for apoptosis-inducing factor (AIF) increase under all stress conditions, as do transcripts for a homolog of Ciapin1, which protects mammalian cells from apoptosis [39]. Two BAX inhibitor proteins, which are anti-apoptotic in higher eukaryotes [34], have transcripts that strongly increase during DS, 6- to 11-fold; whereas transcripts for three proteins involved in the death process of heterokaryon incompatibility, Tol, Vib1, and Pin-c, are reduced by HS and DS.

### Signaling: Bioinformatics

#### Osmotic Pathway

Transcripts for proteins in the osmotic stress pathway [7] are elevated by HS and DS in both wt and os2. These proteins include the histidine kinase-response regulator (HK/RR) Sln1, as well as other HK/RRs [40], such as Hcp1, Nik2, and a likely HK/RR (S1 Table). However, MAPKKK Os4 mRNA increases only in os2 during HS and DS. Not surprisingly, proteomics show that os2 is extremely low in Os2 (0.19), which is a truncated protein and likely unstable. os2 is also moderately low in the upstream Os1 histidine kinase (0.90^§^) and in protein phosphatase-2C (0.88*), whose homologs in yeast dephosphorylate Hog1 [41].

#### Cell Wall Integrity Pathway

Unlike the osmotic stress pathway, transcripts for proteins of the cell wall integrity (CWI) pathway [2, 42] are downregulated during stress, except for wt during DS where they are unchanged (S2 Table). These changes result in 4-fold lower levels of MAPKK Mek1 and MAPK Mak1 transcripts in os2 than in wt during DS. By proteomics (Table 1), os2 also has lower amounts of Mek1 (0.80), as well as lower quantities of Lsp1 (0.47) and Pil1 (0.68), eisosome components [43] that are downstream of Pkc in the yeast CWI pathway. Reduced accumulation of three proteins in the CWI pathway suggests that this pathway may be downregulated in os2, relative to wt, during DS. It also suggests that endocytosis involving eisosomes may be less active in os2 than in wt.

#### Adenylate Cyclase

Intracellular cAMP, produced from ATP by adenylate cyclase, is the downstream second messenger that responds positively to glucose availability [44]. mRNA for the activating cyclase-associated protein (CAP) is lower in os2 during DS than in wt (S2 Table). In contrast, transcripts for the high affinity cyclic nucleotide phosphodiesterase, Acon2, increase only in os2 during DS and are 3.4-fold higher than in wt (S1 Table). Together with lower CAP transcripts, this suggests that os2 may have less active adenylate cyclase and lower levels of cAMP than wt in response to DS.

#### Calcium

Calcium is taken up from the environment or released from organelles in response to stress, so that it accumulates transiently in the cytoplasm [45]. Equilibrium is restored by subsequent calcium efflux out of cells or back into organelles, mediated by calcium-transporting ATPases [46]. Both stresses lead to increased mRNA for calcium-transporting ATPase 3 mRNA in os2 and wt (S1 Table). Proteomics (Table 1) show that ATPase 3 (1.60) and a closely related ATPase (1.21) are elevated in os2. However, the Camk4 calcium/calmodulin-dependent kinase, which has been implicated in stress responses in *Neurospora* [47], is much lower (0.43) in os2 than in wt, suggesting a possible interaction between Os2 and Camk4. As a precedent, the Os2 homolog in *Schizosaccharomyces pombe*, Sty1, is apparently required for transcription of its *camk4* homolog, *srk1* [48]; and Srk1 is phosphorylated by Sty1 [49].

#### Cross Pathway Control

Under conditions of amino acid deficiency or other stresses, changes occur in translation that are mediated by eIF2α kinase (Cpc3/Gcn2) [50–51]. During DS transcripts for this kinase increase in os2 to a level that is 2.7-fold higher than in wt (S1 Table). In the absence of stress, the cross pathway transcriptional program is repressed by Cpc2 [52], which is also part of a quality control complex that inhibits translation of stalled polysomes [53]. Due to the stable level of Cpc2 mRNA in wt during DS, unlike its strong decline during HS, this mRNA is 4.7-fold lower in os2 during DS than in wt (S2 Table). Proteomics (Table 1) also show that os2 is reduced in the repressing Cpc2 (0.81) during DS. Lower expression of the cross pathway control pathway by wt during DS may contribute to its difficulty in translating particular classes of mRNAs during DS and suggests a lack of stress perception by wt.

### Sulfur, Nitrogen, and Lipid Metabolism: Bioinformatics

#### Sulfur

Sulfur-containing compounds are vulnerable to oxidative stress, potentially becoming reactive species themselves, but they are also the chief means by which cells combat oxidative stress [54]. The metabolism of sulfur, therefore, is likely very important under oxidative stress conditions. Sulfur availability appears to be more important for wt during DS than during HS, with transcripts for Cys3, a positive regulator of sulfur acquisition genes [55], increasing only in wt, 9.5-fold, during DS (S1 Table). wt transcripts that remain at control levels during DS, despite being strongly reduced during HS, encode two sulfate permeases, sulfate adenylyltransferase, methionine and cysteine biosynthetic enzymes, and enzymes of the methyl transfer cycle (S2 Table).

Furthermore, proteomics indicate that pathway enzymes for methionine biosynthesis [56–57] are consistently higher in wt than in os2. These include *O*-acetylhomoserine (thiol) lyase (0.89^§^) and three methionine synthases (0.90, 0.89, and 0.45), as well as enolase-phosphatase E-1 (0.79) in the salvage pathway. Also higher in wt are enzymes that participate in the methyl cycle, such as *S*-adenosylmethionine synthase (0.90), methylenetetrahydrofolate reductase (0.83), and serine hydroxymethyltransferase (0.88). os2, instead, has higher amounts of several enzymes that liberate sulfur oxides from organic molecules, in response to sulfur limitation (Table 1). os2 is also higher in enzymes that metabolize hydrogen sulfide, a signaling molecule with cell-protective, anti-oxidant effects [58]. These enzymes include cystathionine γ-lyase (1.20) and sulfide:quinone oxidoreductase (1.10).

#### Nitrogen

Assimilation of nitrogen appears to be more important for both strains during DS than during HS, with transcripts remaining at control levels during DS, rather than showing the sharp declines of HS (S2 Table). Transcripts that show this pattern encode TamA, a positive co-regulator of nitrogen-repressible genes [59], nitrate and nitrite reductase, and the anabolic Am glutamate dehydrogenase. The opposite transcriptional pattern characterizes catabolic enzymes involved in nitrogen acquisition, such as glutamate dehydrogenase 1 and glutaminase A, where increases during HS are much larger than in response to DS (S1 Table). Transcripts for several transporters of nitrogenous compounds are uniquely induced in os2 during DS, including purine permease, the urea/polyamine active transporter, and Nit10 nitrate permease.

As with sulfur assimilation, os2 is lower than wt in enzymes involved in nitrogen assimilation (Table 1), such as nitrate reductase (0.77), nitrite reductase (0.82), and formamidase (0.60). Also lower in os2 are glutamate and glutamine metabolic enzymes, including two glutamine synthetases (0.84 and 0.79) and two degradative glutamate decarboxylases (0.74 and 0.76*). However, os2 is higher in the detoxifying FMN-dependent 2-nitropropane dioxygenase (1.36).

wt and os2 display distinct differences in their pathways for nitrogen metabolism during DS. While transcript analysis (S1 Table) suggests that purines may be utilized as nutrient sources by both strains during DS, proteomics (Table 1) show that wt is higher than os2 in enzymes of purine degradation, specifically uricase (0.78), and enzymes of purine salvage, such as adenosine kinase (0.87), xanthine phosphoribosyltransferase (0.89^§^), and guanylate kinase (0.88*). On the other hand, os2 is higher during DS in ornithine/urea cycle enzymes, involved in arginine metabolism [57], such as arginosuccinate lyase (1.14) and acetylornithine deacetylase (1.27*). os2 is also particularly enriched during DS in transcripts for arginase and agmatinase (S1 Table). Interestingly, in our untargeted metabolomics study [10], arginine was the one normal metabolite detected that increased significantly, approximately 50%, in os2 during both HS and DS and in wt during HS.

#### Lipids

Although transcripts for ceramide synthases are downregulated in all stress conditions, mRNAs for enzymes involved in fatty acid synthesis remain at control levels in wt during DS, rather than sharply declining as in HS. These stable transcripts encode the cel-1 and -2 subunits of fatty acid synthase, a fatty acid elongase, and several fatty acid desaturases (Fig 4, S2 Table). Transcripts for lipid hydrolases, in contrast, such as triacylglycerol lipase, lipase B, cholinesterase, and neutral ceramidases increase during both HS and DS in both strains (S1 Table).

Glycerol synthesis is a response to hyperosmotic stress that is reported to be regulated by Os2 [7]. Transcripts for the biosynthetic glycerol dehydrogenase 1 are induced by DS in wt only, whereas transcripts for glycerol-degrading enzymes, such as glycerol kinase and glycerol-3-phosphate dehydrogenase increase in both strains during stress (S1 Table). Proteomics (Table 1) show that os2 is lower in biosynthetic glycerol dehydrogenases (0.71, 0.84) and higher in the degradative glycerol-3-phosphate dehydrogenase (1.27).

Bioinformatics data on detoxifying enzymes, vesicular trafficking, polyamines, and cell wall proteins are presented in Supporting Information (S1–S3 Tables).

### Western Blot Analysis of Signaling

**Fig 6:**
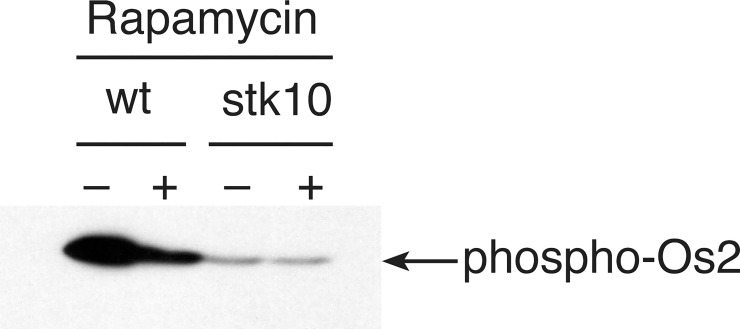
Differential activation of Os2 in Western blot of proteins from wt and stk10 cell extracts. The cells were subjected to DS for 10 min in the presence (+) or absence (-) of rapamycin. Proteins were separated by SDS-gel electrophoresis, and the blot was probed with antibody against mammalian (Thr180/Tyr182) phospho-p38, which detects phosphorylated Os2.

**Fig 7:**
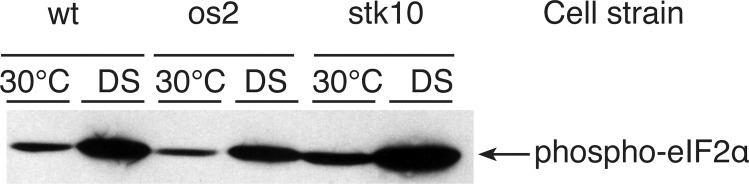
Phosphorylation of eIF2α in response to DS. The cells were either incubated only at 30°C or subjected to DS for 2 hr. Proteins in cell extracts were separated by SDS-gel electrophoresis, and the blot was probed with monoclonal antibody against human (Ser51) phospho-eIF2α, which detects the *Neurospora* homolog.

Since the *stk10* mutant strain, like os2, shows strong resistance to DS, we asked whether it activates Os2 in response to stress. By Western blotting we found that stk10 reproducibly fails to phosphorylate Os2 in response to DS over a low control level (Fig 6, S1 Fig). Since Tor1 directly phosphorylates and activates Sch9, the Stk10 homolog of yeast, we assayed the effect of inhibiting TORC1 by adding rapamycin to wt cells during DS; and we found that rapamycin depressed Os2 phosphorylation (Fig 6, S1 Fig). Typically, stresses like nutrient deficiency and heat shock also result in phosphorylation of the eIF2α translation initiation factor [50]. Western blots showed that phosphorylation of eIF2α occurs in wt, os2, and stk10 in response to DS, and eIF2α remains phosphorylated for at least two hr of DS (Fig 7, S2 Fig). This similarity of the two mutant strains to wt suggests that Os2 and Stk10 do not influence this activation step in cross-pathway control, despite bioinformatics data sugesting that this pathway is depressed in wt, relative to os2, during DS.

**Fig 8:**
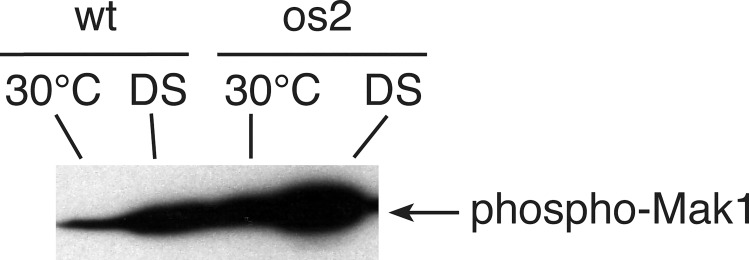
Activation of Mak1 in Western blot of wt and os2 cell extracts. The cells were either incubated only at 30°C or subjected to DS for 10 min. Proteins in cell extracts were separated by SDS-gel electrophoresis, and the blot was probed with monoclonal antibody against human (Thr202/Tyr204) phospho-p44/42 MAP kinase (Erk1), which detects phosphorylated Mak1.

We found that phosphorylation of Mak1, the putative CWI MAP kinase, increased in both wt and os2, as well as other tested mutant strains, subjected to DS (Fig 8, S3 Fig). Neither the *mak1* nor *mek1* mutant strain had detectable phosphorylated Mak1 (S3 Fig). The level of phosphorylated Mak1 is greater in os2 than in wt at both 30°C and DS, suggesting that its hyperactivation might be responsible for os2 resistance to DS. However, we did not find evidence to support this possibility. Addition of a diacylglycerol analog, which should stimulate the CWI pathway, had no effect on DS survival of wt. In addition, both wt and os2 show increased expression of cell wall proteins in response to DS, according to transcript and protein data; and this increase is similar for the two strains (S1–S3 Tables). Furthermore, bioinformatics data suggest that signaling components of the CWI pathway are downregulated in os2 compared with wt.

### Transmission Electron Microscopy

Gene expression and protein data suggest that autophagy is a prominent feature of the response of os2 to DS, and cell survival studies lend support to the cell protective effect of autophagy. To learn more directly whether autophagy likely is occurring in os2 cells during DS, we prepared cells of both strains exposed to control, HS, or DS conditions. We examined thin sections of these cells by transmission electron microscopy (TEM), which has been effectively employed to visualize electron dense autophagic bodies in fungal vacuoles/lysosomes [60].

**Fig 9:**
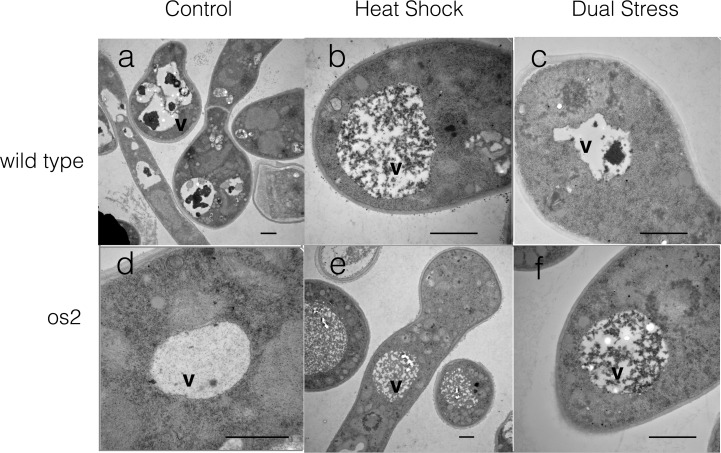
Vacuoles of germinating conidiospores of wt and os2 in transmission electron micrographs. Cells were incubated under 30°C control conditions (a + d) or subjected to heat shock (b + e) or dual stress (c + f) for 2 hr. The vacuoles (lysosomes) are light-colored and indicated by v. The horizontal bar in each micrograph indicates 1 micrometer.

As shown in Fig 9, vacuoles of os2 cells subjected to DS are filled with what appear to be autophagic bodies. Similar autophagic vesicles fill the vacuoles of os2 and wt cells subjected to HS. However, such vacuoles are uncommon in wt during DS, and they contain fewer vesicles. Instead, there are large dark bodies in many of the wt vacuoles (Fig 9), which may be a normal feature of *Neurospora* [61] and seemingly unrelated to autophagy. In wt cells incubated only at 30°C there are similar large, dark bodies evident in the vacuoles, along with vacuoles containing sparse vesicles. In fact, the DS and control vacuoles of wt look very similar to each other. os2 cells that have been exposed only to 30°C have emptier vacuoles than they do during HS or DS, and any vesicular material within them appears diffuse. These micrographs provide evidence that autophagy is likely a prominent feature of the heat shock response of both *Neurospora* strains and that it also characterizes the response of os2 to DS, but autophagy is not characteristic of the wt DS response, where vacuoles appear unchanged from 30°C. Autophagy, therefore, appears to be a feature of the protective heat shock response that is compromised in wt by DS.

## Discussion

We found that the combination of heat stress and nutritional deprivation, induced by a non-metabolizable glucose analog, was lethal to wt cells, while it was well tolerated by a mutant strain lacking a functional stress signaling kinase. Bioinformatic approaches increased our understanding of how the lethal response of wt to DS differs from its protective HS response, and how the *os2* mutant strain differs in its DS response from wt. We have also employed a variety of experimental approaches that reveal many differences between the two strains, some of which likely are relevant to the question of lethality or survival. The question itself is perplexing, since the stress MAP kinase that is nonfunctional in the mutant strain is normally activated by stress and, although non-essential, contributes to a strong HS response (this study, [7]) and would be expected to be beneficial in wt cells.

What seems clear is that the HS response of wt is stronger than that of the *os2* signaling mutant, both in the stress-specific transcripts that are induced to higher levels and in the normal transcripts that are dramatically reduced in wt. When carbohydrate deficiency is added to heat stress, both wt and os2 increase their stress-specific transcripts, with wt achieving very high levels. However, wt deviates from the typical HS pattern in that normal transcripts are only moderately reduced or not reduced at all from 30°C control levels. This inconsistency in wt suggests that it may be invoking two different response patterns to the combined stresses and that these responses conflict. Alternatively, its positive stress response may be so intense under DS that it is unable to respond appropriately to other signals. Despite its strikingly higher amounts of Hsp transcripts, wt accumulates lower amounts of Hsps during DS in comparison to os2. Future experiments may show whether the extremely high mRNA levels of wt for both stress-induced proteins and normal proteins are due to a lack of RNA degradation and whether the low levels of corresponding proteins are due to inefficient translation of these mRNAs or to extensive proteolysis.

Carbohydrate metabolism is altered at the transcript level by both HS and DS, which lead to increased transcripts for enzymes involved in fermentation, an important means for generating energy under stress conditions when oxidative respiration is minimized. In the glycolytic pathway, os2 is higher in enzymes of the ATP-producing stages of the pathway and wt is higher in enzymes of the ATP-utilizing stages. Transcripts for gluconeogenic enzymes increase during stress, and os2 is higher in the rate-limiting enzyme PEP carboxykinase. Overall, os2 appears to be more active in gluconeogenesis and in catabolism for generating glucose and TCA intermediates during DS, as evidenced by its enrichment in enzymes of glycogen degradation and ketone body metabolism. wt, on the other hand, is likely more active in anabolic metabolism, including the pentose phosphate pathway [20], which produces ribose sugars for nucleotides and NADPH for biosynthetic pathways. Subunits of mitochondrial respiratory complexes and their assembly proteins are more abundant in os2 during DS, despite higher wt transcripts for these subunits. Furthermore, proteins that guide mitochondrial fission and morphology are upregulated in os2 over wt during DS, implying greater dynamism in mitochondria of the mutant strain.

ROS are implicated in the lethality of DS, since addition of the anti-oxidant glutathione rescues wt cells from death. In fact, we found that DS, but not HS, leads to increased ROS and very high NO in intact wt cells. In contrast, DS does not lead to increased ROS in os2 and the resultant NO levels are only moderately high. Nevertheless, intrinsic anti-oxidant capabilities do not appear sufficient to explain these differences between wt and os2, since most catalases and thiol-related anti-oxidant proteins are lower in os2 than in wt. Furthermore, according to our recent LC-MS metabolomics analysis [10], glutathione content increased 5-fold (p value of 0.00638) in wt cells subjected to DS. Intracellular ROS are a by-product of oxidative respiration under normal conditions, and mitochondrial ROS increase further when the electron transport chain is inhibited by stress or mutation [23]. The low level of ROS in os2 may be due, at least in part, to its DS-induced increase in alternative mitochondrial electron carriers that circumvent the respiratory complexes where ROS are primarily generated [24].

Many vacuolar/lysosomal proteases are more abundant in os2 during DS than in wt, leading us to believe that autophagy may be an important factor in os2 survival, a proposal supported by further experiments. Our analysis also shows that many transcripts for proteins involved in autophagosome formation and vacuolar biogenesis are upregulated by HS, but only in os2 are they induced to higher levels by DS. Both strains have increased transcripts related to protein ubiquitination and proteasome biogenesis during DS. Transcripts associated with programmed cell death also increase to higher levels during DS than during HS. wt proved to have higher quantities of oxidoreductases that are reported to be pro-apoptotic, whereas os2 had higher quantities of anti-apoptotic oxidoreductases. This balance of pro- and anti-apoptotic proteins supports the possibility, proposed by us earlier [4], that apoptosis or PCD may be responsible for the DS-induced death of wt.

Although DS leads to activation of MAP kinases in two stress pathways, these pathways show divergent transcriptional responses. Transcripts for the osmotic stress pathway are stable or increase during stress, whereas transcripts for the CWI pathway are downregulated by stress, except for wt during DS. Detected CWI pathway proteins are also higher in wt than in os2 during DS. Nevertheless, more phosphorylated Mak1 of this pathway is evident in os2. Perhaps downregulation of CWI transcripts and proteins in os2 is a feedback mechanism to dampen their hyperactivation during DS. The general amino acid control pathway is upregulated by DS. Transcripts for Cpc2, which is a transcriptional repressor of this pathway, decline during all stress conditions, but remain stable during DS in wt, which also has higher quantities of Cpc2. In parallel, transcripts for eIF2α kinase (Cpc3) increase during stress in os2 to higher levels than in wt, suggesting that cross-pathway control is less effective in wt during DS than in os2. Nevertheless, phosphorylation of eIF2α appeared to occur equally in wt and os2 during the initial stages of DS.

Our survival assays of strains deleted in genes for signaling proteins showed that the *stk10* mutant strain survives almost as well as os2 (Fig 1). The yeast homolog of Stk10, Sch9, is directly phosphorylated by activated Tor1 [62]. When we inhibited TORC1 with rapamycin, we found that wt cells were rescued from DS-induced death and survival of os2 and other assayed mutant strains was enhanced. In contrast, rapamycin decreased the strong resistance of stk10 to DS (Fig 1), likely due to pre-existing ineffectiveness of TORC1 in the stk10 strain. We learned that Stk10 is required for phosphorylation of Os2 in response to DS; and like Os2, it is required for resistance to hyperosmotic stress, as confirmed by us (Fig 1) and others [63]. Furthermore, yeast Sch9, which also is required for hyperosmotic stress resistance, was found to associate with Hog1 during osmotic stress; but it did not directly phosphorylate it [64]. However, in a recent report [65], deletion of *sch9* abolished the Hog1 phosphorylation that was induced by C2-ceramide treatment in Isc1-deficient yeast cells.

Our observations suggest that the DS-induced phosphorylation of Os2 may depend indirectly upon activated TORC1, as well as on Stk10. We earlier reported that Os2 activation appeared greater during DS than during HS [4], and we found here that rapamycin addition during DS depressed Os2 phosphorylation in wt. Overall, these results suggest that TORC1 may remain at least partially activated in wt during DS; and being activated, it may phosphorylate Stk10, which is needed for the subsequent phosphorylation of Os2. Nevertheless, much of the damaging effect of the proposed TORC1 activation can evidently be communicated through the activation of Os2 during DS. It has been reported that the p38 MAP kinase of *Drosophila melanogaster* is necessary for activation of TORC1 [66], and there may be a positive feedback loop in *Neurospora* between Os2 and TORC1. Further experiments are needed to directly address the relationship between TORC1 and Os2 during DS.

Earlier reports have shown that autophagy is required for the lifespan extension that is produced by *tor1* or *sch9* mutation [6]. According to our visualization of vacuoles by TEM, autophagy appears to be a prominent feature of the HS response of wt and os2. However, during DS it remains important only for os2 and appears absent in wt. The mammalian p38 MAP kinase has been reported to have contradictory effects on autophagy, either facilitating it [67] or inhibiting it [68], depending on context. This may apply to Os2, as well, since its activation during HS clearly does not interfere with autophagy. However, more highly activated Os2 during DS appears to inhibit autophagy. In contrast, the *os2* mutant strain, which is able to undergo autophagy, tolerates DS well. It is likely that wt cells, characterized by high levels of ROS, undergo apoptosis or PCD, a possibility that needs to be explored further.

A contributing liability for wt may be its increased anabolic activity, possibly driven by TORC1; this is indicated by its relative abundance of pentose phosphate pathway enzymes and greater quantities of enzymes that assimilate sulfur and nitrogen into metaboIites such as methionine and ammonia, respectively. Furthermore, bioinformatics suggest that the biosynthesis of lipids is higher in wt during DS than in os2. In our earlier study of DS, we found that fatty acid synthesis was necessary for wt death [4]. Blocking this synthesis either chemically or genetically allowed cells to fully survive DS. The anabolic activity of wt would be appropriate under growth conditions when resources are abundant, but it is inappropriate under stress. os2, on the other hand, likely utilizes fatty acid catabolism under DS in part to generate substrates for mitochondrial respiration, as judged by its increased content of enzymes for ketone body metabolism. os2 also has a higher complement of mitochondrial proteins involved directly in electron transport, including those that minimize the generation of ROS.

In this report we have sought to understand the basis for DS lethality to wt cells. In summary, we found that DS causes wt cells to diverge from the characteristics of its protective HS response. The DS response of wt deviates in its stabilization or new synthesis of normal mRNAs, increased ROS and NO, likely increased anabolic activity, and lack of autophagy. The DS response of os2 cells, in contrast, more closely resembles its HS response.

## Materials and Methods

### *Neurospora crassa* Strains

The wt strains used in these studies are *N*. *crassa* OR74A (FGSC #2489 and #987), and the *os2* point mutant strain is UCLA 80A in an SL4 background (FGSC #2238). The gene deletion strains reported in this study are os2A (NCU07024, FGSC #17933), stk10a (NCU03200, FGSC #17939), camk4a (NCU09212, FGSC #11545), mek1a (NCU06419, FGSC #11318), mak1A (NCU09842, FGSC #11320), atg1a (NCU00188, FGSC #17400), and atg7a (NCU06672, FGSC #12317). A and a represent the opposite mating types of *N*. *crassa*.

### Growth Conditions

25 mg conidia were inoculated into 25 ml of liquid Vogel’s medium [69] containing 0.05% glucose in 125 ml flasks. After flasks were incubated with shaking for 5 hr at 30°C, they either remained at 30°C (control), were transferred to a 45°C shaking water bath (HS), or were transferred to 45°C with addition of 0.015% 2-DG (DS). All cells were filtered and rinsed at time of collection.

### 2-DG Uptake

[1,2-^3^H]2-deoxy-D-glucose (MP Biomedicals, Irvine CA)), at 1 μCi/ml, was added to 15 ml of 5 hr germinating conidia upon their transfer to DS conditions. Aliquots were removed after 15, 45, and 75 min of continued incubation. The pelleted cells from 150 μl of culture (in triplicate) were solubilized in BioSol (National Diagnostics, Atlanta, GA), according to the manufacturer’s instructions, and were mixed with scintillation fluid (Bio-Safe II, RPI, Mount Prospect, IL) for counting. The top 150 μl supernatant from 500 μl of pelleted culture were directly mixed with scintillation fluid and counted. The percentage uptake was determined by dividing the average pellet-associated tritium counts by the sum of the pellet-associated and supernatant counts, which together approximated 0.15 μCi.

### RNAseq

For RNA isolation, cells were collected after 2 hr of HS or DS treatment or an additional 1 hr at 30°C, and they were suspended in 1 ml RNAzolRT (Molecular Research Center, Cincinnati) and homogenized in a Bead Beater 2 x 45 sec., with cooling in the interval. The supernatant was separated from cell debris and beads by 2 centrifugations at 12,000 x g, the first for 1 min and the second for 10 min; and the supernatant was frozen at -70°C. The manufacturer’s protocol was followed for mRNA isolation from these extracts, and the isolated RNA was solublized in water and frozen at -70°C.

The University of Minnesota Genomics Center performed procedures for Illumina (San Diego, CA) Next-Generation Sequencing for paired end RNA. The RNA was quantified by a fluorimetric RiboGreen assay and its integrity assessed by capillary electrophoresis. RNA samples were converted to Illumina sequencing libraries with Illumina’s Truseq RNA Sample Preparation Kit, according to the manufacturer’s instructions. Steps included oligo-dT purification of polyadenylated RNA and reverse transcription to create cDNA. The cDNA was fragmented, blunt-ended, and ligated to indexed (barcoded) adaptors. The library was size selected, and size distribution was validated by capillary electrophoresis; quantification was by fluorimetry (PicoGreen) and Q-PCR. Indexed libraries were normalized, pooled, clustered on a flow cell, and loaded onto the HiSeq 2000 sequencing system. Library fragments were resynthesized in the reverse direction and sequenced from the opposite end of read 1 fragment to produce the paired end read 2. Primary analysis and de-multiplexing were performed with Illumina’s CASAVA software 1.8.2. The total read numbers were 29,379,956 (wt_30); 38,445,137 (wt_HS); 33,104,645 (wt_DS); 32,677,066 (os2_30); 25,348,748 (os2_HS); and 24,858,572 (os2_DS).

The FASTQ files were analyzed by Galaxy [70], an open-source, web-based program for intensive data analysis, supported by the University of Minnesota Supercomputing Institute. Quality of sequence data was checked with FastQC and reads were aligned by TopHat to the *Neurospora crassa* 74a_12 supercontigs reference genome [71] and to the transcriptome derived by the Broad Institute (Cambridge, MA). Cufflinks was used to assemble transcripts and estimate normalized counts for gene and transcript expression levels (FPKM). Comparisons between different samples to determine differentially expressed genes/transcripts were made by Cuffdiff. Transcript ratios between treatments or strains were considered significant for p values of ≤0.05. This filter was extended to p values of ≤0.15 for transcript comparisons whose ratios were found significant with greater stringency for other comparisons. All ratio values were ≥log_2_ of 1 or -1 (2-fold).

### iTRAQ

wt conidia (in duplicate) and os2 conidia were collected after 3 hr of DS treatment. Cells were lysed by a BeadBeater (Biospec Products, Bartlesville, OK) in buffer consisting of 6 M urea, 0.2% SDS, 0.5 M triethylammonium bicarbonate (TEAB) and 5 mM Tris (2-carboxyethyl)phosphine HCl (TCEP). The supernatant of a low speed centrifugation (1,000 x g for 5 min) was sonicated, after which the supernatant of a high speed centrifugation (14,000 x g for 15 min) was collected. Protein content was determined with the Bradford reagent [72].

Prior to proteolytic digestion of each sample with trypsin, disulfide bonds were reduced and cysteines were alkylated [73]. The trypsinized samples were applied to a 4 ml Extract Clean C18 SPE cartridge (Grace Davidson), and eluents, dried and reconstituted, were labeled with iTRAQ 8-plex reagent (ABSciex, Framingham, MA) per manufacturer’s protocol. After labeling, the samples were pooled, dried and cleaned with a 3 cc Oasis MCX cartridge (Waters Corporation. Milford, MA). The MCX eluent was split in half and dried.

Peptides were separated by two dimensional Liquid Chromatography-Mass Spectrometry (LC-MS), in which the first dimension was offline and employed either strong cation exchange (SCX) [8] or high pH C18 reversed phase separation [73]. Peptide fractions were dried *in vacuo* and reconstituted in water:acetonitrile:trifluoroacetic acid (98:2:0.1). After Stage Tip purification of the first dimension fractions, they were dried *in vacuo* and resuspended in water:acetonitrile:formic acid (98:2:0.01). 1.5 μg of each peptide fraction were injected onto a capillary LC-MS Velos Orbitrap mass spectrometer system (Thermo Fisher, Inc., Waltham, MA) as described previously [74].

We converted the LC-MS raw datafiles to MGF files as described previously [74], and we combined both sets of data, derived from SCX and C18 high pH fractionation, to a single database search by Protein Pilot™ 4.2 (Sciex, Foster City, CA). We used the Broad Institute *Neurospora crassa* protein database from 9/14/11 (http://www.broadinstitute.org/annotation/genome/neurospora/Blast.html), combined with the contaminants database (http://www.thegpm.org/cRAP/index.html), for a total of 10,044 proteins as the reference database. Protein Pilot™ search parameters were: 4-plex peptide label sample type; cysteine methyl methanethiosulfonate; trypsin; instrument Orbi MS (1–3ppm) Orbi MS/MS; biological modifications ID focus; thorough search effort; detected protein threshold 0.05 (10%), competitive error margin 2.00 and false discovery rate analysis invoked (with reversed database). We generated peptide and protein summary exports from Protein Pilot™ and the output was organized in Excel. Where the p value threshold was extended from 0.05 to 0.10 and 0.20, this is indicated in the text by * (0.05 to 0.10) and ^§^ (0.10 to 0.20).

### Cell Survival Measurements

Petri plate assays were performed as described previously [4]. Briefly, conidiospores were incubated in liquid Vogel’s medium for 2 hr at 30°C. Spore suspensions, diluted 5,000-fold in 10% Vogel’s salts, were spread on solidified agar medium containing 0.05% glucose and 0.015% 2-DG. The plates were incubated for 44 hr at 45°C, and the colonies growing from surviving spores were counted. A minimum of three plates was used for each treatment. Additions to the liquified agar medium, for individual experiments, included reduced L-glutathione, probucol, menadione, chloroquine, tunicamycin (all from Sigma-Aldrich, St. Louis), hydrogen peroxide (Fisher Scientific, Pittsburgh), rapamycin (LC Laboratories, Woburn, MA), and 1,2-dioctanoyl-sn-glycerol (Cayman Chemical, Ann Arbor, MI).

### Measurements of ROS and RNS

#### ROS measurement

Ten μM 2’,7’-dichlorodihydrofluorescein diacetate (Cayman Chemical, Ann Arbor, MI) in ethanol were added to 10 ml cultures at time of stress and to the 30°C control. Cells were collected after 1 hr, washed with 1/10 PBS, pH 7.2, resuspended in 1 ml PBS, diluted (1:0.5, 1:1, 1:2, and 1:5) with buffer, and pipetted into microplates. Procedures were performed with minimal light exposure. The fluorescence signal was read from above by a Biotek Synergy MX (Winooski, VT) scanning microplate fluorometer, with excitation and emission wavelengths set at 502 nm and 523 nm, respectively. We programmed an endpoint read at normal speed with automatic sensitivity adjustment. Gen5 software was employed for operations and data analysis.

#### NO measurement

Conidia were cultured as for ROS measurements. After their transfer to treatment conditions, 4-amino-5-methylamino-2’,7’-difluorofluorescein diacetate (DAF-FM diacetate, Life Technologies, Grand Island, NY) in DMSO was added to 1.8 μM final concentration during the last 30 min of a 60 min incubation. The cells were collected, treated, and diluted as for the ROS measurements. For fluorescence scanning, the excitation and emission wavelengths were set at 495 nm and 515 nm, respectively.

### Western Blots

Protein extraction, gel electrophoresis, and Western blotting procedures were carried out as described previously [4]. Primary antibodies (Cell Signaling Technology, Danvers, MA), along with their concentrations, were phospho-p38 MAPK antibody, #9211 (1:1,000); phospho-p44/42 MAPK mAb, #4370 (1:2,000 or 1:3,000); and phospho-eIF2α mAb, #3398 (1:1,000). The secondary antibody (1:20,000) was peroxidase-conjugated goat anti-rabbit IgG (Jackson ImmunoResearch, West Grove, PA).

### Transmission Electron Microscopy

*Neurospora* cultures, transferred from 30°C, were subjected to HS or DS for 2 hr, and the control remained at 30°C for an hr after transfer. To stabilize autophagic vesicles from proteolysis [60], 2 mM phenylmethanesulfonyl fluoride (Sigma-Aldrich, St. Louis, MO) was added from a 100 mM ethanolic stock solution during the last hr of incubation. Cultures were filtered, washed with 10 mM phosphate buffer, pH 7.2, and resuspended in 2.5% glutaraldehyde in 0.1 M phosphate buffer, pH 7.2. Fixation was initiated by exposure of the resuspended cells to vacuum for 15 min at RT and continued overnight with constant mixing at 4°C. Samples were rinsed 3x in 0.1 M phosphate buffer and placed in 1% osmium tetroxide, 0.1 M phosphate buffer overnight at 4°C. Specimens were rinsed in water and embedded in 2% low melting point agarose (Invitrogen, Carlsbad, CA). The samples were cut into 1-mm^3^ pieces and dehydrated in an ethanol series (2x15 min in 25%, 50%, 75%, 95%, and 3x15 min in 100%). Following dehydration the samples were infiltrated with Embed 812 resin (Electron Microscopy Sciences, Hatfield, PA), with the following concentrations: 50% ethanol:50%resin, 100% resin without accelerator, and 100% resin with accelerator (2x), for a duration of 8 hr to overnight at RT for each mix. Specimens were polymerized at 60°C for 48 hr. Ultrathin sections 80–100 nm thick were cut on a Leica Ultracut UCT microtome with a diamond knife and collected on formvar/carbon-coated 200-mesh copper grids. Sections were post-stained with 3% uranyl acetate for 20 min, followed by Sato’s triple-lead stain [75] for 3 min, and examined with an FEI/Philips (Hillsboro OR) CM12 transmission electron microscope operating at 60 kV. Images were recorded with a Maxim DL digital capture system.

## Supporting Information

S1 Fig Original blot for Fig 6: Differential activation of Os2 in Western blot of proteins from wt and stk10 cell extracts. (PDF)

S2 Fig Original blot for Fig 7: Phosphorylation of eIF2α in response to DS. (PDF)

S3 Fig Original blot for Fig 8: Activation of Mak1 in Western blot of wt and os2 cell extracts, left four lanes. The right eight lanes show phosphorylated Mak1 at 30°C (C) and DS (S) conditions for four additional strains: camk4, lsp1, mek1, and mak1.
(PDF)

S1 Table Log_2_ Upregulated RNA Ratios. (PDF)

S2 Table Log_2_ Downregulated RNA Ratios. (PDF)

S3 Table iTRAQ Ratios os2/wt DS. (PDF)
