# Supplementary figures and images for: Glucose Starvation Alters Heat Shock Response, Leading to Death of Wild Type Cells and Survival of MAP Kinase Signaling Mutant
Source: PLoS One. 2016 Nov 21;11(11):e0165980. doi: 10.1371/journal.pone.0165980 (PMC5117620; doi:10.1371/journal.pone.0165980)

MW

97k

66k

45k

30k

20k

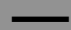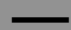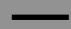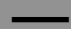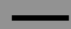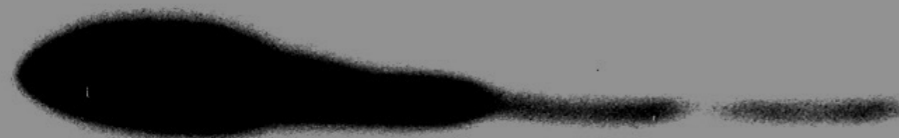

Supplement: S1 Fig — (PDF) [file pone.0165980.s001.pdf]

MW

97k —

66k —

45k —

30k —

20k —

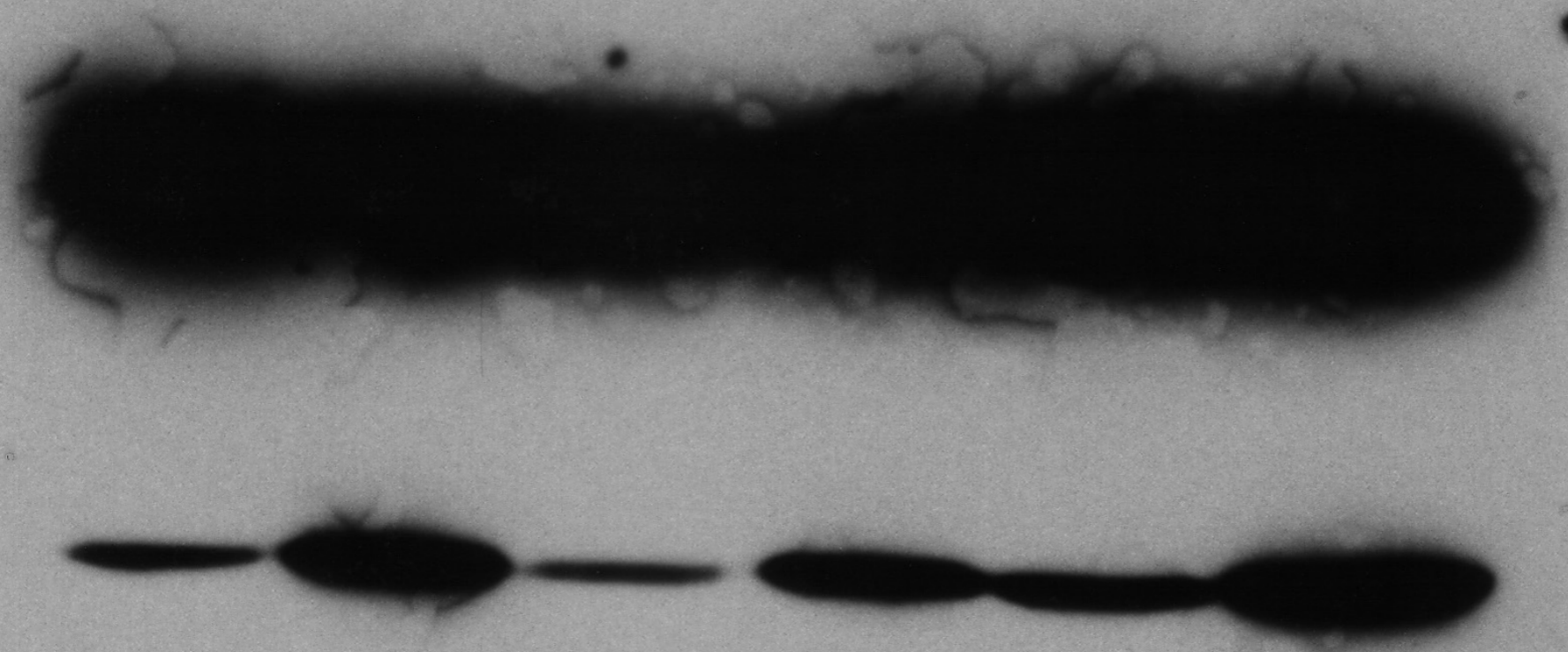

Supplement: S2 Fig — (PDF) [file pone.0165980.s002.pdf]

Fig. S3

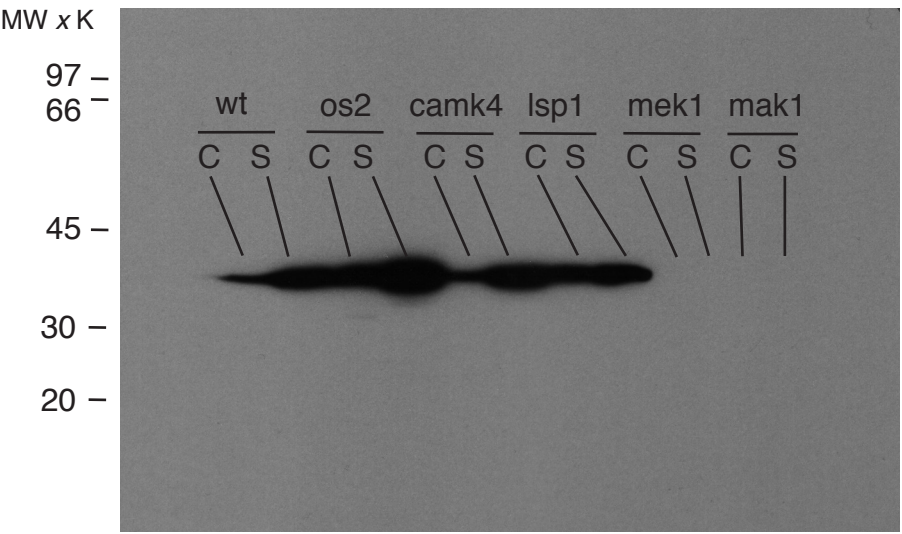

Supplement: S3 Fig — The right eight lanes show phosphorylated Mak1 at 30°C (C) and DS (S) conditions for four additional strains: camk4, lsp1, mek1, and mak1. (PDF) [file pone.0165980.s003.pdf]
