# Supplementary material for: Glucose Starvation Alters Heat Shock Response, Leading to Death of Wild Type Cells and Survival of MAP Kinase Signaling Mutant
Source: PLoS One. 2016 Nov 21;11(11):e0165980. doi: 10.1371/journal.pone.0165980 (PMC5117620; doi:10.1371/journal.pone.0165980)
Supplement: S1 Table — (PDF) [file pone.0165980.s004.pdf]

S1 Table. Log$_2$ Upregulated RNA Ratios

NCU#	Encoded Protein	WT						OS2						DS	
	**STRESS PROTEINS**	HS/30	p val	DS/HS	p val	DS/30	p val	HS/30	p val	DS/HS	p val	DS/30	p val	os2/wt	p val
NCU09364	Hsp30	**7.551**	0.026	----	----	**10.427**	0.000	**6.010**	0.034	**3.109**	0.004	**9.122**	0.001	----	----
NCU02630	Hsp78	**4.445**	0.032	----	----	**8.120**	0.001	**2.721**	0.007	**3.410**	0.001	**6.133**	0.008	----	----
NCU00104	Hsp98	**3.167**	0.132	**5.439**	0.011	**8.541**	0.000	**2.386**	0.042	**3.507**	0.015	----	----	----	----
NCU04172	Fes1 Hsp70 NEF	----	----	**4.801**	0.039	**5.454**	0.000	----	----	**1.780**	0.010	**2.913**	0.013	**-2.712**	0.058
NCU03732	Sis1	**3.309**	0.098	----	----	**5.762**	0.001	**2.592**	0.021	----	----	**3.600**	0.025	----	----
	**GLYCOLYSIS + GLUCONEOGENESIS**														
NCU02702	Glucokinase	**2.496**	0.096	----	----	**2.179**	0.026	**1.409**	0.124	----	----	----	----	**-1.368**	0.087
NCU09873	PEP carboxykinase	**4.383**	0.016	**-2.606**	0.043	**1.716**	0.089	**2.248**	0.032	----	----	**1.734**	0.143	----	----
NCU02906	NAD-dependent malic enzyme 1	**4.106**	0.021	**-2.331**	0.034	----	----	----	----	----	----	----	----	----	----
NCU04797	Fructose Bisphosphatase	**2.326**	0.110	----	----	----	----	----	----	----	----	----	----	----	----
	**PENTOSE PHOSPHATE PATHWAY**														
NCU07143	6-Phosphogluconolactonase	**5.542**	0.018	**-2.550**	0.030	**2.930**	0.071	----	----	----	----	**2.628**	0.106	----	----
	**KETONE BODIES**														
NCU05419	HMG-CoA lyase	----	----	----	----	----	----	**1.650**	0.081	----	----	**1.797**	0.104	----	----
	**FERMENTATION**														
NCU02850	Alcohol dehydrogenase	**3.154**	0.082	**-2.476**	0.045	----	----	----	----	----	----	**2.789**	0.020	----	----
NCU08402	Zinc-binding Adh	**2.575**	0.090	----	----	**3.595**	0.002	**2.530**	0.012	----	----	**2.618**	0.020	----	----
NCU01754	Adh1	**4.401**	0.080	----	----	**4.638**	0.009	**3.197**	0.039	----	----	**4.127**	0.037	----	----
NCU02712	Acetate kinase	**4.246**	0.015	**-2.585**	0.024	**1.598**	0.157	**2.200**	0.079	----	----	**2.108**	0.125	----	----
NCU02397	Ilv5 Pyruvate decarboxylase	**5.421**	0.010	**-2.544**	0.059	**2.815**	0.015	**2.816**	0.017	----	----	**3.232**	0.016	----	----
	**GLYCOGEN**														
NCU07027	Glycogen phosphorylase	----	----	----	----	**1.754**	0.062	**2.292**	0.020	**-1.168**	0.074	----	----	----	----
NCU01517	Gla1 Glucoamylase precursor	**3.268**	0.099	----	----	**1.674**	0.080	**3.034**	0.004	----	----	**2.866**	0.015	----	----
	**MITOCHONDRIA**														
NCU03296	F1 ATPase assembly protein 11	----	----	----	----	**2.324**	0.037	----	----	----	----	----	----	**-1.368**	0.141
	**ALTERNATIVE OXIDOREDUCTASES**														
NCU04874	Aod3 Alternative oxidase	**2.336**	0.149	----	----	----	----	**2.212**	0.063	**2.012**	0.003	**4.228**	0.004	**3.597**	0.001
NCU05225	Nde1 NADH DH	----	----	----	----	----	----	----	----	**1.873**	0.107	----	----	----	----
	**OXIDATIVE STRESS**	HS/30	p val	DS/HS	p val	DS/30	p val	HS/30	p val	DS/HS	p val	DS/30	p val	DS/wt	p val
NCU08791	Catalase 1	**5.310**	0.006	----	----	**3.602**	0.003	----	----	**1.630**	0.027	**1.891**	0.153	**-2.621**	0.003

NCU05770	Peroxidase/catalase 2	**6.641**	0.018	----	----	**6.970**	0.000	**5.439**	0.002	----	----	**5.913**	0.004	----	----
NCU09560	Superoxide Dismutase	**2.715**	0.101	----	----	**2.803**	0.007	**1.575**	0.083	----	----	**2.766**	0.023	----	----
NCU09040	Mig4 oxidoreductase	**6.175**	0.004	----	----	**4.646**	0.001	**3.672**	0.002	----	----	**4.586**	0.001	----	----
NCU03369	Mig8 Pirin	**2.978**	0.054	----	----	**3.621**	0.001	----	----	**1.540**	0.018	**2.811**	0.013	----	----
NCU01759	Mig5 aldo-keto reductase	**3.109**	0.054	----	----	**1.637**	0.086	----	----	----	----	----	----	**-1.934**	0.022
NCU03714	Thioredoxin	**2.556**	0.101	----	----	**3.654**	0.001	**2.255**	0.019	----	----	**2.763**	0.018	----	----
	**AUTOPHAGY + VACUOLES**														
NCU00188	Atg1	**2.127**	0.155	----	----	----	----	**1.245**	0.064	**1.245**	0.064	**2.649**	0.024	**1.622**	0.065
NCU08766	Atg17	**4.638**	0.027	----	----	**2.512**	0.012	**2.460**	0.011	**1.065**	0.114	**3.528**	0.005	----	----
NCU03441	Atg18	----	----	----	----	----	----	----	----	**0.901**	0.157	**1.702**	0.106		
NCU09220	Mon1 Vacuolar fusion protein	**2.779**	0.070	----	----	**1.552**	0.115	----	----	**1.795**	0.007	**2.924**	0.011	**1.297**	0.104
NCU06435	Vps9 Vacuolar sorting	----	----	----	----	----	----	----	----	**0.991**	0.119	**2.156**	0.055	**1.515**	0.062
NCU08761	Vacuolar sorting receptor	----	----	----	----	----	----	----	----	**1.129**	0.077	----	----	**1.533**	0.060
NCU08110	Vacuolar transp chaperone 4	----	----	----	----	----	----	----	----	**1.834**	0.008	----	----	----	----
NCU06231	Vacuolar amino acid transp 1	**2.402**	0.124	----	----	**1.761**	0.058	----	----	**2.349**	0.002	**3.022**	0.021	**1.526**	0.112
NCU07894	Oligopeptide transporter 2	----	----	----	----	----	----	**2.053**	0.019	----	----	**1.797**	0.156	**1.900**	0.091
NCU09278	Env9-like oxidoreductase	----	----	----	----	----	----	**2.646**	0.001	----	----	**3.142**	0.040	**1.841**	0.045
NCU09195	Vacuolar Gpr6	----	----	----	----	----	----	----	----	**1.771**	0.008	----	----	**1.533**	0.060
	**UBIQUITIN + PROTEASOMES**														
NCU05995	Polyubiquitin	----	----	----	----	**3.657**	0.057	----	----	**2.263**	0.048	----	----	----	----
NCU06815	Ubiquitin ligase E3	**2.391**	0.104	----	----	**4.161**	0.000	----	----	**2.473**	0.001	**3.090**	0.012	----	----
NCU06782	Ubiquitin/metalloproteinase	----	----	**2.111**	0.051	**4.279**	0.002	----	----	**1.959**	0.006	**3.043**	0.020	**-1.418**	0.072
NCU01640	Rpn-4 26S proteosome su	**4.179**	0.018	----	----	**3.858**	0.001	**2.181**	0.026	**1.238**	0.059	**3.423**	0.004	----	----
NCU11215	RING-10 ubiquitin hydrolase	----	----	**2.706**	0.021	**3.188**	0.004	----	----	**1.954**	0.005	**1.796**	0.116	**-1.720**	0.036
NCU05582	Ubiquitin fusion degradation	----	----	**1.758**	0.117	**2.002**	0.037	----	----	**1.607**	0.014	----	----	----	----
NCU06856	Ubiquitin fusion degradation	**2.332**	0.119	----	----	**1.491**	0.109	----	----	**1.850**	0.005	**2.185**	0.046	----	----
	**PROGRAMMED CELL DEATH**														
NCU05850	AIF reductase	**2.975**	0.067	----	----	**1.933**	0.080	**2.029**	0.062	----	----	**2.003**	0.101	----	----
NCU02463	BAX-Inhibitor family	----	----	----	----	**3.512**	0.001	**1.681**	0.067	**0.968**	0.140	**2.652**	0.020	----	----
NCU10028	BAX-Inhibitor family	----	----	----	----	**3.221**	0.009	**1.723**	0.145	----	----	**2.773**	0.068	----	----
NCU04315	Ciapin, Fe/S cluster assembly	**2.420**	0.104	----	----	**2.928**	0.005	**1.868**	0.051	----	----	**2.718**	0.019	----	----
	**SIGNALING**														
NCU04615	Sln1	----	----	----	----	**2.985**	0.005	**1.550**	0.095	----	----	**1.882**	0.087	----	----
NCU03071	Os4 MapKKK	----	----	----	----	----	----	**1.300**	0.138	----	----	**1.954**	0.071	**1.151**	0.150
NCU07221	Hcp RR A	----	----	**2.488**	0.082	**3.661**	0.001	----	----	**1.831**	0.007	**2.967**	0.011	----	----
NCU01833	Nik2	**3.151**	0.043	----	----	**2.561**	0.011	**1.240**	0.156	**0.966**	0.124	**2.210**	0.041	----	----
NCU09520	Histidine kinase RR	----	----	**4.678**	0.013	**4.261**	0.000	----	----	----	----	**1.590**	0.130	**-2.090**	0.045
NCU05049	Dual specificity phosphatase	**5.714**	0.009	----	----	**4.678**	0.008	**3.892**	0.009	**1.074**	0.101	**4.968**	0.004	----	----
NCU07495	Lsp1	**3.626**	0.031	----	----	**2.199**	0.030	**1.433**	0.125	**0.924**	0.148	**2.359**	0.038	----	----
NCU00478	Acon2 phosphodiesterase	----	----	----	----	----	----	----	----	**1.322**	0.042	**2.136**	0.053	**1.753**	0.030

NCU07966	Calcium-transporting ATPase 3	**3.050**	0.076	----	----	**3.087**	0.005	----	----	**2.874**	0.000	**3.801**	0.015	----	----
NCU01187	Cpc3	----	----	----	----	----	----	**1.587**	0.083	----	----	**1.578**	0.138	**1.423**	0.085
	**DETOXIFICATION**														
NCU11395	S-OHmethylglutathione DH	**6.620**	0.003	----	----	**4.423**	0.003	**2.722**	0.052	----	----	**3.815**	0.016	**-1.503**	0.069
NCU01127	GSH-dep formaldehyde-activ	**6.270**	0.009	**-2.249**	0.040	**3.959**	0.017	**2.193**	0.132	----	----	**2.450**	0.110	**-1.469**	0.099
NCU09570	Glutathione S-transferase 3	**2.580**	0.160	----	----	**3.139**	0.044	**4.439**	0.008	----	----	**2.779**	0.080	----	----
NCU05780	Glutathione S-transferase 1	----	----	----	----	**1.703**	0.091	----	----	----	----	----	----	**-1.971**	0.021
NCU10521	Glutathione S-transferase 4	----	----	----	----	----	----	**3.173**	0.002	----	----	**2.222**	0.048	----	----
NCU02124	Dienelactone hydrolase	**3.998**	0.018	----	----	**1.882**	0.055	----	----	----	----	----	----	**-1.966**	0.018
NCU07127	Dienelactone hydrolase	----	----	----	----	----	----	----	----	**1.508**	0.029	**1.656**	0.145	----	----
NCU02179	D-Lactate dehydrogenase	**3.559**	0.039	----	----	**2.108**	0.106	**2.349**	0.050	----	----	**2.090**	0.105	----	----
NCU03813	Formate dehydrogenase	**5.224**	0.0079	**-2.384**	0.0616	**2.778**	0.014	**2.249**	0.040	----	----	**2.461**	0.0449	----	----
NCU07322	Glyoxalase	**4.352**	0.014	----	----	----	----	**2.203**	0.055	----	----	**2.095**	0.099	----	----
	**SULFUR + IRON METABOLISM**														
NCU03536	Cys3	----	----	----	----	**3.257**	0.008	----	----	----	----	----	----	**-2.106**	0.122
NCU06041	Aryl sulfatase 1	**3.137**	0.124	----	----	**4.056**	0.038	----	----	**1.667**	0.036	**2.303**	0.128	----	----
NCU06625	Cysteine dioxygenase	**2.070**	0.148	----	----	----	----	**2.170**	0.023	----	----	**2.015**	0.064	**1.301**	0.101
NCU10020	Methionine synthase	**2.703**	0.112	----	----	----	----	**1.388**	0.133	----	----	**2.159**	0.053	----	----
NCU06616	SAM-dep methyltransferase	**3.063**	0.059	----	----	**2.516**	0.035	----	----	----	----	**2.153**	0.091	----	----
NCU00829	Ferric reductase	----	----	----	----	----	----	**2.228**	0.033	----	----	----	----	**2.064**	0.024
NCU00876	Ferric reductase component	**3.161**	0.050	----	----	**1.778**	0.112	----	----	----	----	----	----	----	----
NCU05778	Iron-sulfur cluster assembly 1	----	----	----	----	**1.818**	0.069	----	----	----	----	**1.707**	0.138	----	----
	**NITROGEN METABOLISM**														
NCU04158	Nmr	----	----	----	----	----	----	**1.785**	0.055	----	----	----	----	----	----
NCU00461	Glutamate dehydrogenase 1	----	----	----	----	**1.414**	0.141	**4.257**	0.047	----	----	**2.014**	0.093	----	----
NCU03648	Glutaminase A	**3.957**	0.039	**-2.450**	0.090	**1.445**	0.123	**4.001**	0.001	----	----	**2.997**	0.014	----	----
NCU01106	L-Amino acid oxidase	**4.321**	0.023	**-2.435**	0.036	----	----	----	----	----	----	----	----	----	----
NCU07309	Guanine deaminase	----	----	**2.798**	0.018	**2.928**	0.016	----	----	**1.922**	0.017	----	----	**-2.708**	0.004
NCU02296	Allantoinase 1	----	----	----	----	**1.922**	0.045	----	----	**1.933**	0.005	**2.139**	0.056	----	----
NCU02084	Arginase	----	----	----	----	----	----	----	----	**1.936**	0.008	**2.025**	0.109	**2.268**	0.014
NCU11320	Agmatinase	----	----	----	----	----	----	**1.649**	0.115			**2.403**	0.045	**2.417**	0.008
NCU01348	Agmatinase	----	----	----	----	----	----	----	----	**3.609**	0.003	**5.528**	0.134	**3.772**	0.009
	**LIPIDS**														
NCU10506	Fgl2 Triacylglycerol lipase	**4.162**	0.019	----	----	**2.345**	0.028	**2.941**	0.011	----	----	**2.761**	0.024	----	----
NCU04475	Lipase B	**3.932**	0.025	----	----	**2.592**	0.045	**2.915**	0.028	----	----	**3.321**	0.020	----	----
NCU01747	Gde1	----	----	----	----	----	----	**2.376**	0.019	----	----	**1.771**	0.106	**1.230**	0.134
NCU10400	PldA Phospholipase	**3.312**	0.037	**-1.992**	0.072	----	----	----	----	----	----	----	----	**-2.379**	0.009
NCU09692	Phosphatidic acid phosphatase su	**6.043**	0.004	**-2.655**	0.026	**3.326**	0.016	**2.342**	0.083	**-1.965**	0.013	----	----	**-3.368**	0.001
NCU00292	Cholinesterase	**3.070**	0.066	**-2.254**	0.050	----	----	**2.960**	0.026			**3.139**	0.029	**1.796**	0.046
NCU02070	Pex2	----	----	----	----	----	----	----	----	**1.224**	0.062	**1.885**	0.090	----	----

NCU08058	3-Hydroxyacyl-CoA DH	**2.730**	0.110	----	----	----	----	----	----	**1.212**	0.089	**3.031**	0.041	**1.261**	0.154
NCU04721	Neutral ceramidase			----	----	----	----	----	----	**2.666**	0.000	**1.978**	0.078	**2.034**	0.018
NCU10359	Neutral ceramidase	**6.108**	0.010	----	----	**3.977**	0.016	----	----	----	----	**2.663**	0.090	**-1.427**	0.108
NCU04923	Glycerol dehydrogenase-1			----	----	**1.754**	0.064	**-2.068**	0.037	**2.090**	0.003	----	----	**-1.910**	0.028
NCU04510	Glycerol dehydrogenase-3	**3.583**	0.052	----	----	**1.603**	0.090	**2.197**	0.021	----	----	**1.676**	0.121		
NCU06005	Glycerol kinase	**2.372**	0.135	----	----	**2.512**	0.012	**2.351**	0.016	**-1.305**	0.049	----	----	**-1.765**	0.046
NCU05454	Glycerol 3-P dehydrogenase	**2.937**	0.094	----	----	----	----	**2.481**	0.015	----	----	**1.682**	0.110	----	----
	**VESICULAR SORTING**														
NCU03889	SNARE	----	----	----	----	**2.474**	0.058	----	----	**2.101**	0.021	**2.224**	0.140	----	----
NCU07023	LAlv9 family	----	----	----	----	**1.559**	0.092	----	----	**1.427**	0.026	**1.600**	0.131	----	----
NCU10034	Get1 retrograde vesicle transport	----	----	----	----	**1.525**	0.100	----	----	**1.380**	0.040	**2.413**	0.036	----	----
NCU09432	SNARE-dependent exocytosis	----	----	----	----	----	----	----	----	**1.281**	0.046	**2.380**	0.031	**1.223**	0.125
	**CELL WALL**														
NCU05268	Chitin synthase-6	**5.939**	0.004	----	----	**6.020**	0.000	**4.201**	0.004	**1.823**	0.007	**6.028**	0.000	----	----
NCU12033	Class III chitinase	**4.311**	0.023	----	----	**3.293**	0.023	**3.216**	0.032	**1.679**	0.016	**4.899**	0.007	----	----
NCU08907	cCg13/Non-anchored cell wall 7	**7.368**	0.006	**-3.482**	0.006	**3.824**	0.015	**5.221**	0.030	**1.861**	0.008	**7.086**	0.031	**1.241**	0.134
NCU07817	Non-anchored cell wall-3	**3.113**	0.049	----	----	**3.338**	0.002	**3.123**	0.007	**1.592**	0.017	**4.717**	0.001	----	----
NCU00247	Chitin synthesis regulation	**6.467**	0.014	----	----	**5.742**	0.008	**2.622**	0.098	**3.026**	0.000	**5.652**	0.012	----	----
NCU04189	Cot2	----	----	----	----	**1.834**	0.056	----	----	**2.088**	0.003	**1.747**	0.115	----	----
	**CELL CYCLE**														
NCU00472	Cdc37, Hsp90-binding	----	----	----	----	**2.273**	0.027	----	----	----	----	**1.786**	0.126	----	----
