# Supplementary material for: Glucose Starvation Alters Heat Shock Response, Leading to Death of Wild Type Cells and Survival of MAP Kinase Signaling Mutant
Source: PLoS One. 2016 Nov 21;11(11):e0165980. doi: 10.1371/journal.pone.0165980 (PMC5117620; doi:10.1371/journal.pone.0165980)
Supplement: S2 Table — (PDF) [file pone.0165980.s005.pdf]

S2 Table. Log$_2$ Downregulated RNA Ratios

NCU#	Encoded Protein	WT						OS2						DS	
	**RIBOSOMES**	HS/30	p val	DS/HS	p val	DS/30	p val	HS/30	p val	DS/HS	p val	DS/30	p val	os2/wt	p val
NCU06661	60S ribosomal protein L22	**-5.944**	0.055	**3.626**	0.019	**-2.381**	0.054	**-3.330**	0.134	----	----	**-4.477**	0.062	**-1.813**	0.058
NCU05599	40S ribosomal protein S28	**-4.974**	0.056	**3.873**	0.028	----	----	**-2.938**	0.102	----	----	**-3.159**	0.086	**-1.761**	0.092
NCU09094	Ipi-1 pre-rRNA-processing	**-3.445**	0.048	**3.488**	0.006	----	----	----	----	**-1.818**	0.011	**-1.907**	0.116	**-1.554**	0.072
	**GLYCOLYSIS**														
NCU02252	Emp6 Phosphoglycerate mutase	**-4.397**	0.019	**3.846**	0.006	----	----	**-1.998**	0.052	----	----	**-2.086**	0.070	**-1.705**	0.047
NCU09489	Phosphoglycerate mutase	----	----	----	----	**-2.027**	0.094	----	----	**-1.708**	0.057	**-2.976**	0.046	----	----
NCU00720	Tca17 L-lactate dehydrogenase	**-3.450**	0.034	**3.974**	0.004	----	----	----	----	----	----	**-1.604**	0.125	**-2.245**	0.009
	**PENTOSE PHOSPHATE PATH**														
NCU06142	Transaldolase	----	----	----	----	----	----	**-3.676**	0.008	----	----	**-2.411**	0.051	**-2.211**	0.013
	**TCA CYCLE**														
NCU06482	Ace2 pyruvate DH E1 α su	**-3.339**	0.080	**3.264**	0.038	----	----	----	----	----	----	----	----	----	----
NCU03004	Ace3 pyruvate DH E1 comp	**-3.968**	0.036	**3.321**	0.013	----	----	----	----	----	----	----	----	----	----
NCU01692	Citrate synthase	**-3.149**	0.124	**2.897**	0.068	----	----	**-1.924**	0.111	----	----	----	----	**-1.489**	0.148
NCU04899	Tca15 Malate dehydrogenase	**-3.006**	0.096	**4.010**	0.035	----	----	----	----	----	----	----	----	----	----
	**MITOCHONDRIA**														
NCU01808	Cyc1 Cytochrome c	**-4.067**	0.086	**5.165**	0.051	----	----	----	----	----	----	----	----	----	----
NCU03340	Cytochrome-c oxidase su VIIc	**-2.088**	0.156	**3.267**	0.025	----	----	----	----	----	----	----	----	**-1.927**	0.035
NCU06695	Cytochrome c oxidase su 6	----	----	**2.438**	0.056	----	----	----	----	----	----	----	----	**-1.589**	0.070
NCU01606	ATP synthase su 5	**-2.707**	0.108	**2.755**	0.045	----	----	----	----	----	----	----	----	----	----
NCU16844	Cytochrome b-c1 complex su	**-2.518**	0.116	**3.654**	0.030	----	----	----	----	----	----	----	----	**-1.814**	0.075
NCU09816	Cyt26 b-c1 complex	**-3.244**	0.072	**2.737**	0.042	----	----	----	----	----	----	----	----	**-1.282**	0.146
NCU02217	Cox23 Cyt c oxidase assembly	----	----	----	----	**-2.317**	0.042	----	----	----	----	----	----	----	----
	**ALTERNATIVE OXIDOREDUCT**														
NCU07953	Aod-1	----	----	----	----	----	----	----	----	**-2.174**	0.002	----	----	----	----
	**OXIDATIVE/NITRO STRESS**	HS/30	p val	DS/HS	p val	DS/30	p val	HS/30	p val	DS/HS	p val	DS/30	p val	os2/wt	p val
NCU10051	Yhb1-like NO reductase	**-2.930**	0.081	**3.049**	0.036	----	----	**-2.644**	0.017	**2.348**	0.001	----	----	----	----
	**AUTOPHAGY**														
NCU06666	Inositol 3-phosphate synthase	**-4.431**	0.045	**4.2047**	0.022	----	----	**-2.428**	0.053	----	----	----	----	**-1.819**	0.085
	**PROGRAMMED CELL DEATH**														
NCU04452	Mig3/Oye2 NADPH2 DH	----	----	**2.279**	0.048	----	----	**-1.543**	0.093	**1.106**	0.090	----	----	**-1.202**	0.128

NCU03725	Vib1	**-2.699**	0.066	**2.658**	0.017	----	----	----	----	----	----	**-1.626**	0.1186	**-1.428**	0.075
NCU03494	Pinc	**-4.125**	0.041	**2.928**	0.027	----	----	**-5.582**	0.021	----	----	**-4.335**	0.016	**-2.838**	0.011
NCU04453	Tol	----	----	----	----	**-1.572**	0.1373	**-2.414**	0.036	----	----	**-2.491**	0.045	----	----
	**SIGNALING**														
NCU09842	Mak1 MAPK	**-4.117**	0.030	**4.346**	0.009	----	----	**-1.594**	0.142	----	----	----	----	**-2.069**	0.036
NCU06419	Mek1 MAPKK	**-2.628**	0.106	**1.8114**	0.112	----	----	**-2.034**	0.031	----	----	**-2.290**	0.038	**-2.021**	0.016
NCU08008	Cyclase-associated protein CAP	----	----	**2.657**	0.037	----	----	----	----	----	----	----	----	**-1.843**	0.038
NCU06948	Calmodulin-like EF-hand	**-2.848**	0.145	**3.563**	0.016	----	----	**-2.713**	0.041	----	----	----	----	**-2.471**	0.012
NCU05810	Cpc2	**-5.106**	0.083	**4.199**	0.034	----	----	**-2.832**	0.104	----	----	**-2.918**	0.102	**-2.236**	0.064
	**DETOXIFICATION**														
NCU00028	S-(OHmethyl)glutathione DH	**-2.664**	0.076	----	----	----	----	**-1.862**	0.045	----	----	**-1.985**	0.067	**-1.985**	0.017
NCU01157	Glu-Cys ligase catalytic su	**-2.338**	0.110	----	----	----	----	**-1.560**	0.086	----	----	**-2.510**	0.029	**-1.382**	0.093
	**SULFUR METABOLISM**														
NCU06055	Extracellular alkaline protease	**-4.679**	0.013	**3.267**	0.005	**-1.474**	0.116	**-5.235**	0.000	----	----	**-4.246**	0.001	**-3.028**	0.001
NCU03247	TauD	**-3.083**	0.053	----	----	**-1.959**	0.057	**-3.612**	0.007	----	----	**-3.901**	0.006	**-2.109**	0.025
NCU01057	TauD	**-3.475**	0.081	**4.114**	0.007	----	----	**-1.921**	0.124	**1.500**	0.046	----	----	----	----
NCU04433	Cys14 sulfate permease II	**-3.580**	0.035	**1.860**	0.101	**-1.782**	0.072	**-2.714**	0.009	----	----	**-3.217**	0.009	----	----
NCU03235	Sulfate permease II	**-2.416**	0.118	----	----	**-1.844**	0.080	**-2.864**	0.018	----	----	**-3.202**	0.018	**-1.513**	0.096
NCU01985	Cys11 Sulfate adenylyltransf	**-4.420**	0.021	**2.707**	0.021	**-1.774**	0.071	**-3.234**	0.007	----	----	**-3.349**	0.010	**-1.571**	0.054
NCU02005	P-adenosine P-sulfate reductase	----	----	----	----	----	----	----	----	**2.113**	0.012	----	----	----	----
NCU02430	Met7 Cystathionine γ-synthase	----	----	----	----	----	----	----	----	**-1.469**	0.022	**-2.223**	0.046	**-1.179**	0.134
NCU08434	Met6	**-4.057**	0.024	**3.492**	0.008	----	----	**-2.282**	0.021	----	----	**-2.539**	0.026	**-2.332**	0.008
NCU09230	Cys16 Cystathionine γ-lyase	**-2.177**	0.150	**1.977**	0.073	----	----	**-1.930**	0.048	----	----	**-1.818**	0.099	----	----
NCU02657	Eth1 S-adenosylmeth synth	**-4.034**	0.062	**3.317**	0.034	----	----	**-2.152**	0.099	----	----	**-2.329**	0.101	**-1.699**	0.086
	**NITROGEN METABOLISM**														
NCU05994	TamA	----	----	**1.749**	0.103	----	----	**-2.064**	0.036	**1.508**	0.026	----	----	**-1.115**	0.151
NCU05298	Nit3 nitrate reductase	**-3.081**	0.066	**2.179**	0.057	----	----	**-3.454**	0.002	**3.337**	0.000	----	----	----	----
NCU04720	Nit6 nitrite reductase	**-5.224**	0.011	**4.712**	0.001	----	----	**-6.042**	0.000	**4.726**	0.000	----	----	----	----
NCU01066	L-Amino acid oxidase	**-3.047**	0.082	**1.982**	0.107	----	----	**-2.497**	0.045	**3.072**	0.000	----	----	**1.824**	0.038
NCU01195	Am Glutamate dehydrogenase	**-5.765**	0.103	**5.613**	0.042	----	----	**-5.202**	0.060	**3.242**	0.000	----	----	----	----
NCU03257	Mep1 Ammonium transporter	**-2.328**	0.134	**1.889**	0.101	----	----	**-2.029**	0.041	**1.211**	0.061	----	----	----	----
NCU01065	Mep2 Ammonium transporter	----	----	----	----	**-2.466**	0.107	**-5.105**	0.007	**4.094**	0.000	----	----	----	----
NCU07334	Uracil permease	----	----	----	----	----	----	**-2.391**	0.026	**3.315**	0.000	----	----	----	----
NCU09909	Urea active transporter	**-2.564**	0.109	**1.990**	0.078	----	----	**-1.972**	0.104	**4.995**	0.000	**3.026**	0.011	**2.576**	0.004
NCU07205	Nit10 Nitrate permease	----	----	**2.245**	0.066	----	----	**-1.565**	0.081	**3.466**	0.000	**1.904**	0.126	----	----
NCU06918	Purine permease	----	----	----	----	----	----	----	----	**2.590**	0.000	**2.456**	0.027	**2.404**	0.005
NCU02629	Ad5 Bifunctional purine biosyn	**-4.988**	0.016	**3.481**	0.004	**-1.569**	0.111	**-2.301**	0.032	----	----	**-3.017**	0.019	**-1.513**	0.062
NCU06187	Ad4 Adenylosuccinate lyase	**-3.663**	0.027	**2.641**	0.019	----	----	----	----	----	----	**-2.715**	0.018	**-1.714**	0.038
NCU09789	Ad8 Adenylosuccinate synthet	**-2.983**	0.080	----	----	**-1.911**	0.049	----	----	----	----	**-1.780**	0.123	----	----
NCU08162	Arg10 Arginosuccinate lyase	**-3.552**	0.043	----	----	**-2.216**	0.024	----	----	----	----	----	----	**1.496**	0.070

NCU07732	Arg2 Carbamoyl-P synthase su	----	----	----	----	**-1.736**	0.072	----	----	----	----	----	----	----	----
NCU01271	Spe1 Ornithine decarboxylase	**-2.719**	0.109	----	----	----	----	----	----	----	----	----	----	----	----
NCU01083	Spe2 S-Adenosylmet decarbox	**-3.326**	0.040	**2.452**	0.031	----	----	**-1.361**	0.121	----	----	**-2.250**	0.044	**-1.235**	0.126
NCU06727	Spe3 Spermidine synthase	**-3.831**	0.056	**2.440**	0.052	**-1.453**	0.158			----	----	**-2.438**	0.074	----	----
NCU03963	Nic7 MTAP	**-4.907**	0.010	**4.276**	0.001	----	----	**-2.138**	0.024	----	----	**-2.536**	0.027	**-1.526**	0.064
	**LIPIDS**														
NCU07308	Cel1 Chain elongation-1	**-4.184**	0.028	**3.236**	0.014	----	----	----	----	**-1.307**	0.046	**-2.150**	0.088	----	----
NCU07307	Cel2 Chain elongation-2	**-4.808**	0.023	**4.171**	0.007	----	----	----	----	----	----	----	----	----	----
NCU08976	Fatty acid elongase	**-4.250**	0.024	**2.932**	0.013	**-1.379**	0.156	**-1.592**	0.109	----	----	**-2.743**	0.021	**-1.620**	0.046
NCU04156	Erg1 C-8 Sterol isomerase	**-4.132**	0.021	**4.266**	0.004	----	----	**-2.405**	0.020	----	----	**-1.652**	0.144	**-1.547**	0.079
NCU03006	Erg4 Sterol 24-C-methyltrans	**-2.770**	0.077	**2.990**	0.020	----	----	**-2.322**	0.017	**1.643**	0.013	----	----	----	----
NCU04461	Erg28-like	**-3.906**	0.087	**2.936**	0.058	----	----	**-2.349**	0.040	----	----	**-2.890**	0.029	**-2.100**	0.034
NCU09497	Bifunctional D12/D15 FA desat	**-6.640**	0.004	**3.861**	0.002	**-2.841**	0.007	**-4.734**	0.001	----	----	**-5.312**	0.001	**-2.475**	0.005
NCU02209	Oleate D12 desaturase	**-5.616**	0.014	**3.714**	0.003	**-1.964**	0.055	----	----	**-1.462**	0.027	**-2.849**	0.034	----	----
NCU02408	Fatty acid desaturase	----	----	----	----	**-1.930**	0.054	----	----	----	----	**-1.810**	0.106	----	----
NCU09858	Acylamide-D3(E)-desaturase	----	----	----	----	----	----	**-3.451**	0.020	----	----	**-3.020**	0.053	**-1.462**	0.138
NCU02468	Lac1 Longevity-assurance prot	**-3.203**	0.049	----	----	**-2.234**	0.022	**-1.647**	0.077	----	----	**-2.453**	0.032	----	----
NCU00008	Lass1 Longevity-assurance prot 1	**-2.902**	0.065	----	----	**-1.820**	0.068	**-1.856**	0.053	----	----	**-2.063**	0.070	----	----
NCU01116	Ceramide glucosyl transferase	----	----	----	----	----	----	----	----	**1.504**	0.031	----	----	**1.290**	0.130
NCU06342	Phospholipase D	**-3.101**	0.048	----	----	**-1.778**	0.061	**-1.749**	0.053	----	----	**-2.194**	0.044	----	----
NCU03068	Pdx3 Glycerol-3-P phosphatase 1	**-2.246**	0.134	**2.743**	0.016	----	----	----	----	----	----	----	----	**-1.210**	0.132
NCU03176	Choline kinase	----	----	----	----	----	----	**-2.113**	0.045	----	----	**-2.615**	0.032	**-1.371**	0.120
	**CELL WALL**														
NCU08457	Eas/Ccg2	**-3.313**	0.052	**3.781**	0.015	----	----	**-6.194**	0.000	**4.176**	0.000	**-2.015**	0.111	**-1.581**	0.089
NCU03013	Anchored cell wall protein 10	**-5.399**	0.014	**5.506**	0.004	----	----	**-4.369**	0.002	**2.170**	0.002	**-2.196**	0.078	**-2.609**	0.019
