# Supplementary material for: Glucose Starvation Alters Heat Shock Response, Leading to Death of Wild Type Cells and Survival of MAP Kinase Signaling Mutant
Source: PLoS One. 2016 Nov 21;11(11):e0165980. doi: 10.1371/journal.pone.0165980 (PMC5117620; doi:10.1371/journal.pone.0165980)
Supplement: S3 Table — (PDF) [file pone.0165980.s006.pdf]

S3 TABLE. iTRAQ Ratios os2/wt DS

NCU#	Protein	os2/wt	p value
	**FERMENTATION**		
NCU02193	Pyruvate Decarboxylase	1.27	0.019
NCU02476	Alcohol DH III	4.59	0.000
NCU09285	Zn-Alcohol DH	1.30	1.82E-07
NCU08402	Zn-Alcohol DH	1.16	0.028
NCU01754	Alcohol DH I	0.87	0.007
NCU04823	NADP-Alcohol DH C	0.52	5.80E-08
	**GLYCOGEN**		
NCU07027	Glycogen Phosphorylase	1.15	0.000
NCU00743	Glycogen Debranching Enz	1.14	0.000
NCU08131	α-Amylase	1.37	0.002
	**DETOXIFICATION**		
NCU04368	Gst3 Glutathione S-transferase	0.75	1.51E-05
NCU04676	Glutathione S-transferase	0.61	0.002
NCU02888	Glutathione Transferase Domain	1.16	0.02
NCU04569	5-Oxoprolinase	0.69	1.85E-11
NCU07153	Glutamate Carboxypeptidase	0.88	0.004
NCU00173	Esterase D	0.89	0.027
NCU06652	S-OH-Methylglutathione DH	0.82	0.014
NCU11395	S-OH-Methylglutathione DH	0.57	1.04E-07
NCU03358	Ketoreductase	0.86	0.019
NCU03749	OH-Acylglutathione Hydrolase	0.86	0.012
NCU04815	Lactoylglutathione Lyase	0.73	0.044
NCU09600	Dienelactone Hydrolase	0.80	0.032
NCU02124	Dienelactone Hydrolase	0.72	1.75E-07
NCU07127	Dienelactone Hydrolase	0.61	0.002
NCU03283	2-Keto-4-Penenoate Hydratase	0.84	0.004
NCU03813	Formate DH	0.91	0.072
NCU05883	Xenobiotic Mono-Oxygenase	3.66	0.097
NCU09741	NADPH-Cytochrome P450 Red	0.82	0.017
NCU06327	Benzoate 4-Monooxygen P450	0.84	0.103
NCU07240	Aldehyde Reductase	0.81	0.001
NCU08882	Dihydrodiol DH	1.16	0.009
	**LIGHT- & CLOCK-REGULATED**		
NCU07787	Ccg14	2.09	0.02
NCU08936	Ccg-15, Acw-1	1.22	0.003
NCU09559	Ccg-9 Trehalose synthase	0.68	0.017
NCU07267	Bli-3, Pyridoxal Phosphate Biosyn	0.65	2.65E-05
NCU03753	Ccg-1 Glucose-Repressible	0.63	9.09E-05
NCU08699	Bli-4	0.42	0.001

NCU#	Protein	os2/wt	p value
	**CYTOSKELETON & VESICLES**		
NCU04244	Syntaxin 2	1.27	0.041
NCU09243	Sec-9-Like	1.22	0.141
NCU08092	Nipsnap Family	1.16	0.172
NCU03387	Sec18	1.11	0.164
NCU05234	Ypt6-Like GTPase	1.13	0.123
NCU07319	COP1 Coatomer β	0.92	0.08
NCU02538	COP1 Coatomer ζ	0.87	0.119
NCU02510	Clathrin Heavy Chain	0.91	0.002
NCU04115	Clathrin Light Chain	0.9	0.182
NCU03800	Endosomal Cargo Receptor	0.82	0.089
NCU01587	Cofilin	0.9	0.057
NCU10777	GMF Family	0.89	0.099
NCU07010	Asp1	0.85	0.154
NCU02455	FKBP22	0.78	0.015
NCU08897	Sec61 α	0.88	0.134
NCU06702	Yop1	0.88	0.105
NCU06361	Reticulon-Like	0.77	0.002
NCU09132	α-Tubulin	0.9	0.022
NCU03306	Tubulin-Folding Cofactor A	0.9	0.176
NCU02459	Memo1 Family	0.81	0.005
	**CELL WALL**		
NCU04883	Chitinase 1	2.41	0.022
NCU06010	Mutanase	1.72	0.027
NCU05404	Endoglucanase	1.6	0.028
NCU07974	Endoglucanase B	1.37	0.002
NCU07253	β-Glucanosyltransferase Gel1	1.4	8.70E-06
NCU07366	Glucosaminefructose 6-P aminotr	1.22	0.003
NCU05974	Glucanosyltransferase Mwg1	1.15	0.009
NCU04189	Cot2	1.2	0.098
NCU05667	Acw3	1.51	0.016
NCU02041	Acw11	1.29	0.103
NCU01902	Glucosamine 6-P N-Acetyltransf	0.85	0.02
NCU04674	α-Glucosidase	0.65	0.008
NCU06871	β-Glucan Synthase Comp Gls1	0.87	0.051
NCU06781	β-Glucanosyltransferase Gel2	0.83	0.049
NCU06850	β-Glucanosyltransferase Gel3	0.64	0.061
NCU03530	Acw6	0.49	0.111
NCU02948	Ncw4	0.58	1.39E-07
